# Tactical Tentacles: New Insights on the Processes of Sexual Selection Among the Cephalopoda

**DOI:** 10.3389/fphys.2019.01035

**Published:** 2019-08-21

**Authors:** Peter Morse, Christine L. Huffard

**Affiliations:** ^1^Australian Institute of Marine Science, Crawley, WA, Australia; ^2^College of Science and Engineering, James Cook University, Townsville, QLD, Australia; ^3^Monterey Bay Aquarium Research Institute, Moss Landing, CA, United States; ^4^California Academy of Sciences, San Francisco, CA, United States

**Keywords:** cryptic female choice, cuttlefish, mate choice, octopus, polyandry, sperm competition, squid, reproduction

## Abstract

The cephalopods (Mollusca: Cephalopoda) are an exceptional class among the invertebrates, characterised by the advanced development of their conditional learning abilities, long-term memories, capacity for rapid colour change and extremely adaptable hydrostatic skeletons. These traits enable cephalopods to occupy diverse marine ecological niches, become successful predators, employ sophisticated predator avoidance behaviours and have complex intraspecific interactions. Where studied, observations of cephalopod mating systems have revealed detailed insights to the life histories and behavioural ecologies of these animals. The reproductive biology of cephalopods is typified by high levels of both male and female promiscuity, alternative mating tactics, long-term sperm storage prior to spawning, and the capacity for intricate visual displays and/or use of a distinct sensory ecology. This review summarises the current understanding of cephalopod reproductive biology, and where investigated, how both pre-copulatory behaviours and post-copulatory fertilisation patterns can influence the processes of sexual selection. Overall, it is concluded that sperm competition and possibly cryptic female choice are likely to be critical determinants of which individuals' alleles get transferred to subsequent generations in cephalopod mating systems. Additionally, it is emphasised that the optimisation of offspring quality and/or fertilisation bias to genetically compatible males are necessary drivers for the proliferation of polyandry observed among cephalopods, and potential methods for testing these hypotheses are proposed within the conclusion of this review. Further gaps within the current knowledge of how sexual selection operates in this group are also highlighted, in the hopes of prompting new directions for research of the distinctive mating systems in this unique lineage.

## 1. Introduction

Sexual selection is the competition within one or both sexes of a species toward optimising individual reproductive success (Darwin, 1906; Bateson, 1983). The resulting disparity in reproductive outcomes among individuals in a species can lead to the development of specific behaviours and/or phenotypic traits that can enable individuals who display them to increase their genetic contribution to subsequent generations (West-Eberhard, 1983; Andersson and Simmons, 2006). Anisogamy, which is the differential investment between males and females toward their gametes in most animal mating systems (Kodric-Brown and Brown, 1987), typically results in conflicting strategies for enhancing reproductive output between males and females of the same species (Chapman et al., 2003). Females, which have a relatively higher investment toward gametes, generally have reproductive capacities that are resource-limited (Bateson, 1983; Kodric-Brown and Brown, 1987). Meanwhile males, which are usually less limited by their gamete production, are primarily limited by the number of female gametes they can successfully fertilise (Kodric-Brown and Brown, 1987). Therefore, where anisogamy exists sexual selection can impose females to evolve mechanisms by which they can obtain more resources to create higher numbers of healthy viable eggs, and/or to fertilise these eggs with sperm from higher quality and/or genetically compatible males (Kirkpatrick, 1982; Kodric-Brown and Brown, 1987; Tregenza and Wedell, 2000; Kokko et al., 2003). Dissimilarly, sexual selection will often drive males of a species to develop traits or behaviours that enable them to achieve copulations with a higher number of females, to mate with healthier more fecund females and to attain greater fertilisation success with the females they mate with (Parker, 1970; Kodric-Brown and Brown, 1987; Reinhold et al., 2002).

The cephalopods (Mollusca: Cephalopoda) are a class of invertebrates that might provide a different type of model for studying the mechanisms and impacts of sexual selection. Spermatozoa of male cephalopods are encased in a finite number of discrete spermatophores that are transferred to the female, in some cases individually (Mann et al., 1970). Depending on species, males may or may not be able to regenerate spermatophores (Anderson et al., 2002). In species where males can regenerate spermatophores, the time and energy needed to do so can potentially limit male mating frequency during a spawning period (Mann et al., 1970; Anderson et al., 2002). The constraint of having a fixed or limited male reproductive capacity might lead to a higher investment by males toward their gametes, and could present a system where both male and female mate selection might be important to the reproductive success of individuals within species (e.g., Huffard et al., 2008, 2010). Additionally, several other cephalopod characteristics make this a unique and interesting class of animals for studying the processes of sexual selection. Male and female promiscuity are reported across this class (Hall and Hanlon, 2002; Hanlon et al., 2002; Huffard et al., 2008; Arnold, 2010; Squires et al., 2013; Morse et al., 2015). Males are known to employ size-conditional mating strategies (Hanlon et al., 1997; Hall and Hanlon, 2002; Huffard et al., 2008; Lin et al., 2018). Females in some species are known to be selective of mates (Hall and Hanlon, 2002; Wada et al., 2005a), can store sperm (Perez et al., 1990; Hanlon et al., 1999; Morse, 2008; Hoving et al., 2010; Bush et al., 2012; Cuccu et al., 2014) and can potentially be selective about which sperm they use (Naud et al., 2004; Shaw and Sauer, 2004; Buresch et al., 2009; Sato et al., 2013). While females of the southern bottletail squid (*Sepiadarium austrinum*) can gain nutritional and likely fecundity benefits through the consumption of spermatophores (Wegener et al., 2013), in most cases females receive no identifiable resources or parental care from the males they mate with. This suggests that male quality and/or genomic compatibility might be important factors in female mate selection, as observed in other animals (Jennions and Petrie, 1997; Tregenza and Wedell, 2000; Kokko et al., 2003). Furthermore, cephalopods' capacity for complex behavioural and visual displays can enable unique modes of courtship and/or discretion of potential mates (Corner and Moore, 1981; Hanlon et al., 1994; Huffard, 2007; Mäthger and Hanlon, 2007; Mäthger et al., 2009).

This review is divided into four broad sections following the introduction. The first of these briefly summarises the present knowledge of how reproduction takes place within each of the nine currently recognised extant cephalopod orders (Allcock et al., 2015; Sanchez et al., 2018). The following section focuses in greater detail on pre-copulatory behaviour observed in three coastal cephalopod families, the loliginid squid (Myopsida: Loliginidae), cuttlefish (Sepiida: Sepiidae) and octopuses (Octopoda: Octopodidae), and specifically addresses the mechanisms and behaviours that might lead to differential copulatory rates within these more thoroughly studied mating systems. The third section summarises recent advances in understanding the post-copulatory processes that might lead to differential male fertilisation success among the five cephalopod families where this topic has been investigated. This review concludes with a final section highlighting some of the gaps in the current knowledge of cephalopod mating systems, and which might serve as feasible and productive topics for investigation in the near future. Biases in coverage by this review reflect the skew of existing behavioural research toward more accessible, abundant, and day-active species instead of offshore, nocturnal, and rarer forms.

## 2. Reproductive Biology of Cephalopods

### 2.1. General Reproductive Strategies and Life History Traits of the Cephalopoda

Shallow-water coastal cephalopods are typically known for having relatively fast growth rates and short life-cycles ended with a terminal spawning season (Joll, 1976; Le Goff and Daguzan, 1991; Jackson, 2004; Sato et al., 2008). By contrast, more protracted spawning periods have been observed in deep-sea and cold-water pelagic taxa which might have longer life spans, and pygmy species which can increase lifetime fecundity through release of multiple clutches (Boletzky, 1986; Rocha et al., 2001). Difficulty in finding mates, small egg-clutches due to limitation of resources and low offspring survival, as well as stable environmental conditions with reduced predation of adults have each been hypothesised as selective pressures toward increased parental investment and multiple spawning events for cephalopod taxa occupying deep-sea habitats (Rocha et al., 2001; Hoving et al., 2015). Some life history characteristics of deep-sea and oceanic cephalopods include relatively longer life-cycles, prolonged embryonic development within larger eggs, maternal care of egg masses, intermittent or continuous spawning over a terminal breeding season and/or iteroparity throughout the adult phase (Villanueva, 1992; Seibel et al., 2000; Rocha et al., 2001; Hoving et al., 2004, 2008, 2015; Barratt et al., 2007; Laptikhovsky et al., 2007, 2008; Arnold, 2010; Bush et al., 2012; Table 1).

**Table 1:** The life history characteristics pertaining to reproductive biology are summarised below for the nine extant orders of Cephalopoda.

**Order**	**Approx. no. of species**	**Size range**	**Lifespan**	**Method of sperm transfer**	**Female sperm storage organ**	**Site of fertilisation**	**Reproductive cycle^[32]^**	**Fecundity**	**Maternal care**	**Hatchling type**
Nautilida	7^[1]^	Shell up to 229 mm in diameter^[1]^	>20 years^[5]^	Spadix^[15]^	Organ of Valenciennes^[15]^	Oocyte micropyles (hypothesised^[28]^)	PS^[28]^	10–20 eggs/year^[40, 41]^	Not reported	Direct developing^[50]^
Oegopsida	236^[2]^	20 to at least 2,000 mm ML^[3]^	Up to at least 2 years^[6]^	Hectocotylus or elongated terminal organ^[16]^	Dorsal pouches^[18]^; inside MC^[19]^; or external^[16]^	Thought to be external^[16]^	MS^[33]^; or ITS^[16]^	Up to 6 million^[16]^	Extended egg care in two species^[45, 46]^	Planktonic larvae^[51]^
Myopsida	50^[2]^	20–900 mm ML^[3]^	1–2 years^[7]^	Hectocotylus^[3]^	Sperm receptacle near BA; or inside MC^[20]^	External^[29]^	ITS^[29]^	~2,000–55,000^[42]^	ANG^[47]^	Planktonic larvae^[51]^
Idiosepiida	6^[2]^	<25 mm ML^[1]^	80–151 days^[8, 9]^	Hectocotylus^[1]^	Sperm receptacle near BA^[21]^	External^[30]^	CS^[34]^	53–922^[34]^	Not reported	Direct developing^[34]^
Sepiolida	70^[2]^	Up to 80 mm ML^[1]^	5 months to reports of 2 years^[10]^	Hectocotylus^[1]^	Internal spermatheca^[22]^; or external^[23]^	External^[22]^; or “confined external”^[31]^	ITS^[22]^; or CS^[35]^	Up to 931^[43]^	ANG^[48]^	Direct developing^[51]^
Sepiida	120^[2]^	60–510 mm ML^[1]^	1–2 years^[11]^	Hectocotylus^[1]^	Paired sperm receptacles near BA; or external^[24]^	External^[22]^	ITS^[36]^	Up to 8,000^[36]^	ANG^[49]^	Direct developing^[51]^
Spirulida	1^[2]^	~45 mm ML^[1]^	18–20 months^[1]^	Hectocotylus^[1]^	Sperm receptacle near BA^[1]^	Unknown	Unknown	Unknown	Not reported	Unknown
Octopoda	300^[2]^	15 mm ML (~1 g) to over 600 mm ML (>180 kg)^[4]^	~7 months^[12]^ to 5 years^[13]^	Hectocotylus in Incirrata^[4]^; Unknown in Cirrata	Oviduccal glands^[25]^; ovaries^[26]^; or inside dismembered hectocotyli within MC^[27]^	Oviduccal glands^[25]^; or inside ovaries^[26]^	STS^[37]^; MS^[38]^; or CS^[39]^	30^[44]^-700,000^[37]^	Extended egg care in Incirrata^[12]^; not reported in Cirrata	Planktonic larvae^[37]^; or direct developing^[12]^
Vampyromorphida	1^[2]^	Up to 130 mm ML^[4]^	Predicted >8 years^[14]^	Funnel (hypothesised^[17]^)	Infraorbital pits^[17]^	Unknown	Suggested to be PS^[14]^	Up to 20,711^[14]^	Not reported	Unknown

*ANG, accessory nidamental gland; BA, buccal area; CS, continuous spawning; ITS, intermittent terminal spawning; MC, mantle cavity; ML, mantle length; MS, multiple spawning; PS, polycyclic spawning; STS, simultaneous terminal spawning*.

1 (Jereb and Roper, 2005);

2 (Allcock et al., 2015);

3 (Jereb and Roper, 2010);

4 (Jereb et al., 2014);

5 (Dunstan et al., 2011);

6 (Hoving and Robison, 2017);

7 (Jackson, 2004);

8 (Tracey et al., 2003);

9 (Sato et al., 2008);

10 (Marine Biological Laboratory, 2019);

11 (Gabr et al., 1998);

12 (Tranter and Augustine, 1973);

13 (Hartwick, 1983);

14 (Hoving et al., 2015);

15 (Mikami and Okutani, 1977);

16 (Hoving et al., 2004);

17 (Pickford, 1949b);

18 (Hoving et al., 2007);

19 (Durward et al., 1980);

20 (Hanlon et al., 1997);

21 (Sato et al., 2010);

22 (Squires et al., 2013);

23 (Hoving et al., 2009);

24 (Naud et al., 2005);

25 (Froesch and Marthy, 1975);

26 (Perez et al., 1990);

27 (Laptikhovsky and Salman, 2003);

28 (Arnold, 2010);

29 (Hanlon et al., 2004);

30 (Sato et al., 2013);

31 (Hoving et al., 2008);

32 (Rocha et al., 2001);

33 (Nesis, 1996);

34 (Nishiguchi et al., 2014);

35 (Laptikhovsky et al., 2008);

36 (Laptikhovsky et al., 2003);

37 (Joll, 1976);

38 (Rodaniche, 1984);

39 (Caldwell et al., 2015);

40 (Okubo et al., 1995);

41 (Uchiyama and Tanabe, 1999);

42 (Hixon, 1980);

43 (Salman and Önsoy, 2010);

44 (O'Dor and Malacaster, 1983);

45 (Seibel et al., 2000);

46 (Bush et al., 2012);

47 (Barbieri et al., 1996);

48 (Collins et al., 2012);

49 (Richard et al., 1979);

50 (Carlson et al., 1992);

51 *(Boletzky, 1987). Taxonomic orders and the sequence they are presented in are based on phylogenies described in Allcock et al. (2015)*.

This latter mode of life history is exemplified by the nautilids (Nautiloidea: Nautilida), which are expected to live more than 20 years and spawn seasonally each year once sexually mature (Mikami and Okutani, 1977; Saunders, 1984; Arnold, 2010; Dunstan et al., 2011). Nautilids are taxonomically distinct from other cephalopods in that they are the only extant representatives of ectocochleate, or externally shelled cephalopods (Cephalopoda: Nautiloidea; Voss, 1977; Sanchez et al., 2018). However, several coleoid taxa (Cephalopoda: Coleoidea) are also reported to spawn over multiple seasons. These taxa include: several oegopsid squids (Decapodiformes: Oegopsida; Harman et al., 1989; Hoving et al., 2004), *Vampyroteuthis infernalis* (Octopoda: Vampyroteuthidae; Hoving et al., 2015), *Opisthoteuthis* spp. (Octopoda: Opisthoteuthidae; Villanueva, 1992), *Graneledone* spp. (Octopoda: Megaleledonidae; Bello, 2006; Guerra et al., 2012), *Octopus chierchiae* (Octopoda: Octopodidae; Rodaniche, 1984) and the currently undescribed larger Pacific striped octopus (“LPSO”; Octopoda: Octopodidae) which has a continuous spawning phase (Caldwell et al., 2015). These taxa, with the exceptions of *O. chierchiae* and LPSO are all either deep-sea or pelagic cephalopods. Some of the larger oegopsid squid, *V. infernalis* and the giant Pacific octopus (*Enteroctopus dofleini*) have relatively slower growth rates and are estimated to live for 2–8 years (Hartwick, 1983; Hoving et al., 2015; Hoving and Robison, 2017). However, the rest of the coleoid cephalopods are thought to have life-spans of only several months to 2 years (Table 1), and in the case of terminal spawners, die shortly after breeding (McGowan, 1954; Roper, 1965; Joll, 1976).

### 2.2. Reproductive Biology in Nautilida

**Figure 1:**
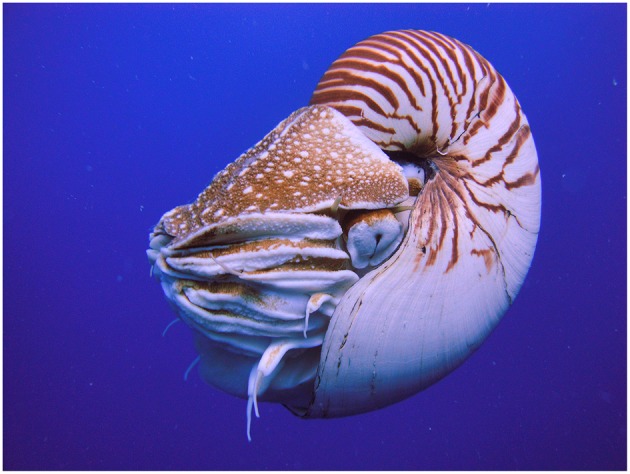
The Palau nautilus, *Nautlius belauensis* (Nautiloidea: Nautilida). Photograph taken by Manuae, and downloaded via Wikimedia under licence: [CC BY-SA 3.0 (https://creativecommons.org/licenses/by-sa/3.0)].

The order Nautilida (Figure 1) contains only two genera, *Allonautilus* and *Nautilus*, and as mentioned above these taxa are the only extant representatives of the cephalopod subclass: Nautiloidea. Correspondingly, their life histories are relatively unique among cephalopods in that they have opportunities to breed continuously throughout their extended life spans (Mikami and Okutani, 1977; Saunders, 1984; Arnold, 2010). Distributional data have indicated that nautilid populations, where sampled, always have an operational sex ratio (OSR) biased toward males (1:3 in Saunders and Ward, 1987). Additionally, Haven (1977) who sampled a population of *Nautilus pompilius* year round to depths of 340 m, found an increase in female catch rates between January and May. These data suggest there might be a seasonal migration of females in this species, possibly related to an annual breeding, feeding, or spawning season.

Relatively very little is known about the reproductive habits of nautilids in the wild. Aquarium observations have provided a basic understanding of copulatory behaviour in captive individuals (*N. macromphalus* in Mikami and Okutani, 1977; *N. pompilius* in Arnold, 2010). Successful copulations takes place by the male grasping the female with his tentacles and drawing the pair's mantle apertures together. The male then uses an enlarged labial tentacle, called a spadix, to push the female's buccal tentacles to the side and transfer one long spermatophore (~30 cm) to the female's organ of Valenciennes (Mikami and Okutani, 1977), an analogue to the seminal receptacle in some coleoid cephalods except that the spermatophores appear to remain intact within the organ of Valenciennes until the time of fertilisation (Arnold, 2010). The exact method of fertilisation is still not understood in nautilids. However, it has been hypothesised that the spermatophore(s) break during egg-laying and the spermatozoa migrate independently toward the oocyte micropyle(s) (Arnold, 2010).

Copulations have been reported to last as long as 30 h (Mikami and Okutani, 1977), and females have been observed as passive throughout the process. Males are frequently observed to bite the females on the mantle and shell during copulation (Arnold, 2010), the reason for which is still not understood. Bites were observed to leave marks on the shell, suggesting that these could theoretically be used as an indication of a female's mating and/or possibly egg-laying history (Arnold, 2010). However, males' response to and/or preference for females with different numbers of bite marks have not been assessed.

Arnold (2010) additionally indicated that male copulation attempts are extended to any object that shares a similar shape and size of another nautilid, and that male/male copulation attempts are common. This suggests that nautilids may have difficulty recognising conspecifics and/or discriminating between sexes. This aspect of social naiveté might be related to living in the pelagic environment where the ability to find mates could be limiting to reproductive success. In this context, it might to be less costly for males to waste time and/or energy attempting unviable copulations, than to risk missing an opportunity to transfer gametes to a suitable mate, as has been hypothesised for other cephalopod species (Hoving et al., 2012; Morse et al., 2015).

In captivity, nautilids deposit eggs both singly and in small clusters on aquarium floors over an extended annual period, and to do so over multiple years (Carlson et al., 1992; Arnold, 2010). The eggs' exteriors are tough, flexible and opaque white in colour (Mikami and Okutani, 1977). Embryonic development in nautilids takes from 9 months to over a year (Arnold, 2010), and there have been no observations of maternal egg care. Upon hatching, juveniles appear like miniature adults and are immediately capable of actively swimming and feeding on cut-up pieces of prawn (Carlson et al., 1992).

### 2.3. Reproductive Biology in Oegopsida

**Figure 2:**
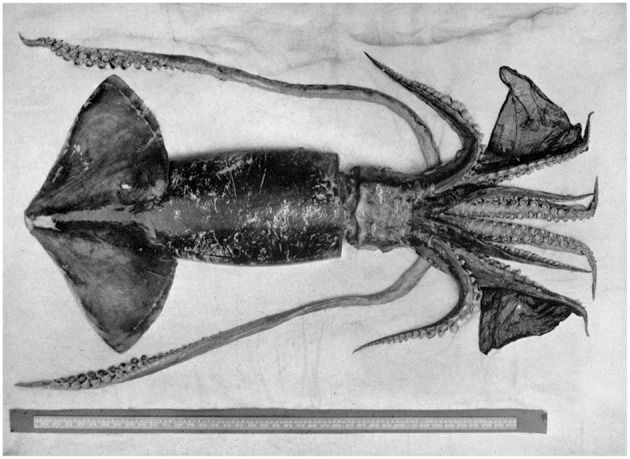
A dead specimen of the neon flying squid, *Ommastrephes bartamii* (Decapodiformes: Oegopsida). Photograph taken by the British Museum of Natural History, and downloaded from the public domain via Wikimedia.

Depending on species, male oegopsid, or “open-eye” squids (Decapodiformes: Oegopsida; Figure 2), are thought to use either a hectocotylised arm or an elongated terminal organ to deliver sperm to the female (Nesis, 1996; Hoving et al., 2004). However, the method and placement of sperm transfer can take place in a variety of ways depending on the taxon. In *Lycoteuthis lorigera*, females can store spermatophores in dorsal pouches located on the neck (Hoving et al., 2007). *Illex* spp. (Oegopsida: Ommastrephidae) are not known to have any seminal receptacle, and sperm are stored only inside spermatophore casings within females' mantle cavities (Durward et al., 1980; Arkhipkin and Laptikhovsky, 1994). External spermatophore placement is also common in several species of deep-sea squids, including *Architeuthis* sp. (Hoving et al., 2004), *Octopoteuthis deletron* (Hoving et al., 2012), *Taningia danaei* (Hoving et al., 2010) and *Moroteuthis ingens* (Hoving and Laptikhovsky, 2007). This method of spermatophore placement has been suggested as a consequence of size dimorphism between the sexes, in that smaller males need to be able to mate quickly and escape from larger and potentially cannibalistic females (Hoving et al., 2004). Where studied, externally placed spermatophores in oegopsid species enter through females' skin autonomously to achieve implantation and storage (Hoving and Laptikhovsky, 2007).

The oegopsids have the highest fecundity among the cephalopod class. Oocyte counts have led to estimations of fecundity reaching as high as 1–6 million in *Dosidicus gigas* (Ehrhardt et al., 1983) and 3- 6 million in *Architeuthis* sp. (Hoving et al., 2004). Both of these deep-water species have extended or multiple spawning events, enabling higher fecundity than their counterpart taxa in coastal or shallow-water habitats (Rocha et al., 2001). Egg deposition and care are variable among the oegopsids. In deep-sea habitats where there are typically few hard surfaces enabling egg attachment, where known, most oegopsids lay eggs in neutrally buoyant egg masses that they let go into the water column (Guerra et al., 2002; O'Shea et al., 2004; Staaf et al., 2008). By contrast, females in the family Enoploteuthidae lay single, buoyant egg-capsules (Young and Harman, 1985). Maternal egg care has been reported in *Bathyteuthis berryi* and *Gonatus onyx*. These species have been known to carry their egg masses in their arms and guard them from predators and parasites throughout their development (Seibel et al., 2000; Bush et al., 2012). In the case of *G. onyx*, embryonic development is estimated to take up to 9 months, and females will drop their two feeding tentacles after egg deposition, presumably to better hold the egg mass with their eight arms (Seibel et al., 2000). Larval morphology is highly variable among oegopsids, however all taxa studied to date are planktonic upon hatching (Boletzky, 1987).

### 2.4. Reproductive Biology in Myopsida

**Figure 3:**
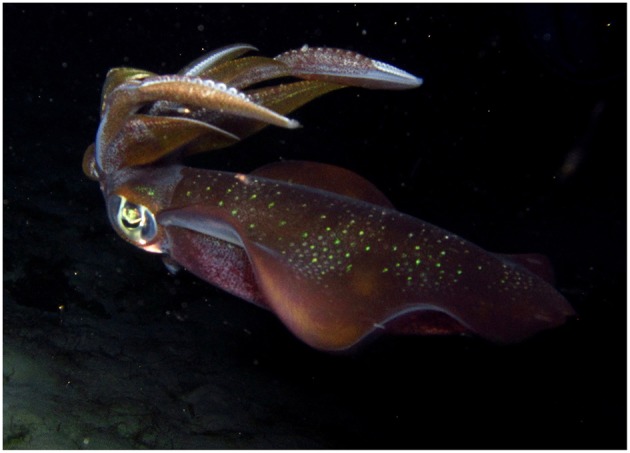
The bigfin reef squid, *Sepioteuthis lessoniana* (Decapodiformes: Myopsida) from Komodo National Park. Photograph by P. Morse.

The myopsid, or “closed-eye” squids (Decapodiformes: Myopsida; Figure 3) are an order of neritic squids that typically live for only 1–2 years (Jackson, 2004), spawn in large assemblages (Hanlon, 1998; Hanlon et al., 2002; Jantzen and Havenhand, 2003) and are thought to reproduce over only one breeding season (McGowan, 1954; Roper, 1965). Males can transfer sperm in at least three ways within myopsid taxa. Typically, pairs can mate in either a head to head or parallel position, and males use their hectocotylised arms to place spermatophores in either the seminal receptacles near the females' buccal mass or inside females' mantle cavities near the distal ends of their oviducts (Hanlon et al., 1997; Jantzen and Havenhand, 2003). Female *Lolliguncula brevis* have specialised pads on the inside of their mantle walls where males place spermatophores during parallel mating (Hanlon et al., 1983). A third method of spermatophore placement has been observed during sneaker copulations (described in section 3.1.1) in *Loligo vulgaris*. Sneaker males in this species have been observed to opportunistically place spermatophores directly into females' arms either on or near eggs that are about to be deposited onto an egg mass (Hanlon et al., 2002). Fertilisation in myopsids is external, and takes place at the time of egg deposition (Hanlon et al., 2004; Shaw and Sauer, 2004; Naud et al., 2016).

Where studied, spawned egg counts in Myopsida have ranged from 2,024 in *L. brevis* to 55,308 in *Doryteuthis pealei* (Hixon, 1980). Myopsids deposit eggs on the substrate, either in single clutches or in communal egg masses (Hanlon et al., 1997; Jantzen and Havenhand, 2003). Female myopsids possess an accessory nidamental gland (ANG) and it is has been hypothesised that bacterial communities, cultured here and passed to egg capsules, might help to protect myopsid eggs from fouling or harmful microbes (Barbieri et al., 1996). All myopsids are intermittent terminal spawners (Roper, 1965; Hanlon et al., 2004), meaning the females deposit eggs in multiple batches over a single spawning period and die shortly after (Rocha et al., 2001). One species, *D. opalescens*, was previously reported to have simultaneous terminal spawning (McGowan, 1954). However, females of this species have since been observed depositing eggs in multiple batches, and to re-join the shoal between discrete egg-laying events (Hanlon et al., 2004). No maternal egg care has been reported within this order (apart from the protective properties imparted by the ANG), and all myopsid larvae are planktonic upon hatching (Boletzky, 1987).

### 2.5. Reproductive Biology in Idiosepiida

**Figure 4:**
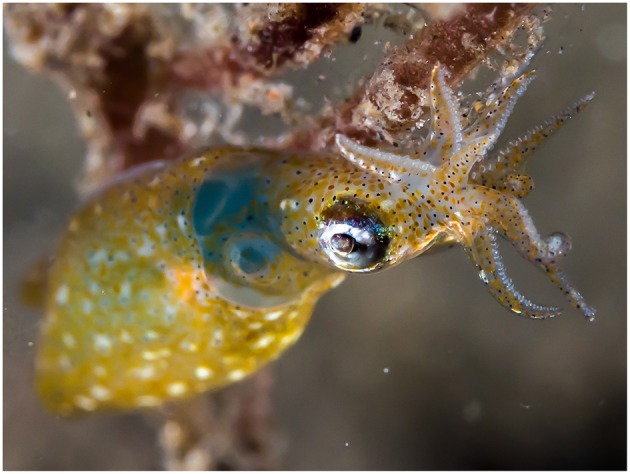
The two-toned pygmy squid, *Idiosepius pygmaeus* (Decapodiformes: Idiosepiida). Photograph taken by krokodiver and downloaded via Flickr under licence: [CC BY-SA 2.0 (https://creativecommons.org/licenses/by/2.0/)].

Pygmy squids (Decapodiformes: Idiosepiida) are an order of small, short-lived (80–150 days: Tracey et al., 2003; Sato et al., 2008), continuous spawning, shallow-water, coastal cephalopods comprising the single genus, *Idiosepius* (Nishiguchi et al., 2014; Figure 4). Copulations in this group take place in a head to head position where the male attaches spermatangia to the base of the female's arms (Kasugai, 2000; Sato et al., 2013). It is thought that the spermatozoa might then actively swim from the spermatangia to the female's seminal receptacle located near the buccal membrane (Sato et al., 2010). Like in myopsids, egg fertilisation in idiosepiids is external and takes place at the time of egg deposition (Sato et al., 2013). In captivity, female *I. pygmaeus* have been observed to lay a total of 53–922 eggs over up to eight egg clutches (Nishiguchi et al., 2014). Eggs are deposited individually into an egg capsule which is attached to the substrate (Natsukari, 1970) and are reported to hatch after approximately 7–40 days of development depending on species (Nabhitabhata et al., 2004; Kasugai and Segawa, 2005). Idiosepiids are direct developing, however all hatchlings are planktonic during their juvenile stage (Nishiguchi et al., 2014).

### 2.6. Reproductive Biology in Sepiolida

**Figure 5:**
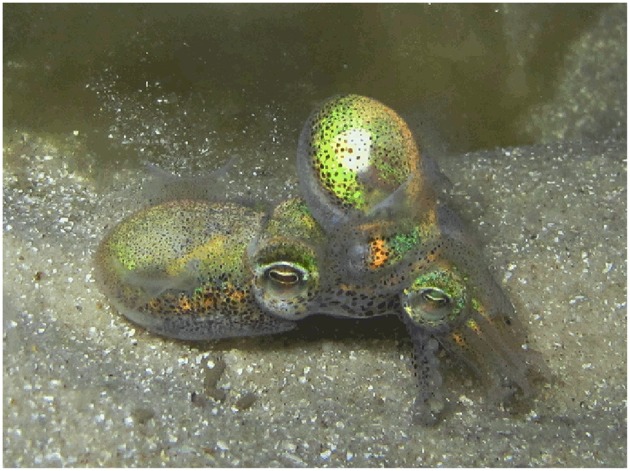
A male (left) southern dumpling squid, *Euprymna tasmanica* (Decapodiformes: Sepolida) grasps the female (right) around the lower mantle during mating. Photograph taken by Zoe Squires, downloaded via Wikimedia and cropped under licence: [CC BY 4.0 (https://creativecommons.org/licenses/by/4.0)].

Similar to other decapod cephalopods (Cephalopoda: Decapodiformes), fertilisation in most bobtail squids (Decapodiformes: Sepiolida) takes place externally (Rodrigues et al., 2009; Squires et al., 2013; Wegener et al., 2013). However in at least one species, the pelagic *Heteroteuthis dispar*, fertilisation has been reported to take place either in the female oviducts or visceropericardio coelom through what the authors refer to as “confined external fertilisation” (Hoving et al., 2008). During copulation, sepiolid males usually use their arms to latch onto females' necks (Rodrigues et al., 2009; Squires et al., 2013; Figure 5), or in the case of *Rossia pacifica* grasp females from a parallel position (Brocco, 1971). In most cases males then use their hectocotylised first left arms to transfer spermatangia to inside females' mantle cavities where sperm is stored in posterior pouch-like receptacles (Hoving et al., 2008; Rodrigues et al., 2009; Squires et al., 2013). *Rossia moelleri* is an interesting exception. Males of this species are known to implant spermatangia into females' external mantle tissue (Hoving et al., 2009). These authors suggest that a combination of mechanical and chemical processes aid the spermatangia to enter through females' skin autonomously to the oviducts where it is hypothesised that fertilisation might occur in this species.

Sepiolids tend to lay comparatively fewer and larger eggs than most other coleoid cephalopods (Rocha et al., 2001; Laptikhovsky et al., 2008; Squires et al., 2013). Fecundity in sepiolids has been recorded up to 646 eggs in captive southern dumpling squid, *Euprymna tasmanica* (Squires et al., 2013), and up to 931 eggs in *R. macrosoma* (Salman and Önsoy, 2010). Maternal care has not been reported among sepiolid taxa, but many female sepiolids are known to disguise their eggs with opaque egg casings, ink or sand (Arnold et al., 1972; Rodrigues et al., 2009; Squires et al., 2013), and to inoculate their eggs through the ANG (Collins et al., 2012). Sepiolids resemble their adult forms upon hatching and lack a planktonic phase (Boletzky, 1987).

### 2.7. Reproductive Biology in Sepiida

**Figure 6:**
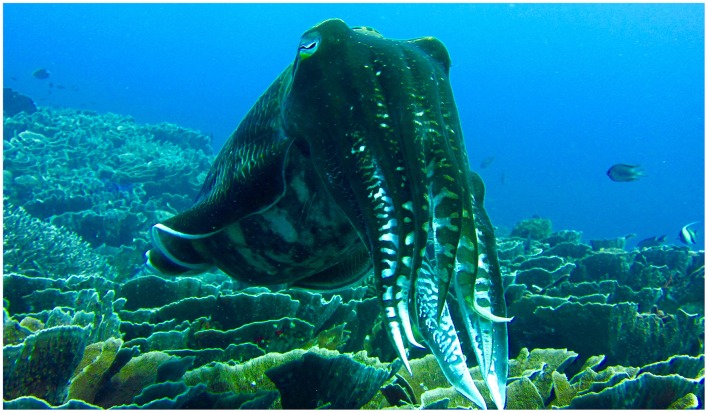
The broadclub cuttlefish, *Sepia latimanus* (Decapoformes: Sepiida) from Komodo National Park. Photograph by P. Morse.

In sepiids (Decapodiformes: Sepiida; Figure 6), fertilisation, where studied, is always external (Naud and Havenhand, 2006). Eggs are fertilised by sperm either stored in females' seminal receptacles, located ventral to their buccal membrane, or from recently deposited spermatophores on females arms and/or buccal areas (Naud et al., 2005). Reproductive behaviour has been recorded in great detail for *Sepia officinalis* and *S. apama* (Hanlon et al., 1999; Naud et al., 2004). Copulations in these species take place in the head to head position. Pairs face each other, intertwine arms and males use their hectocotylised fourth left arms to transfer spermatophores from their funnel to females' seminal receptacles and/or directly onto females' buccal areas. Males then use the hectocotylus to break open spermatophores, and possibly to manipulate their placement on the female. The female then uses either the stored or externally placed sperm to fertilise her eggs individually at the time of deposition (Naud et al., 2005).

Large sepiids are estimated to live from 1–2 years (Le Goff and Daguzan, 1991; Gabr et al., 1998) and are predicted to lay up to 8,000 eggs over intermittent spawning periods (Laptikhovsky et al., 2003). Female sepiids are not known to physically guard their eggs, but similar to sepiolids, several mechanisms for hiding eggs are employed across these taxa. Where observed, female sepiids always attach their eggs to the substrate or a hard object (Adamo et al., 2000; Hall and Hanlon, 2002). *Sepia officinalis* lay opaque eggs, darkened with ink to minimise detection by predators (Boletzky et al., 2006). Female *S. esculenta* achieves the same result by attaching sand and rubble to their eggs with a sticky exterior (Natsukari and Tashiro, 1991). Both *S. latimanus* and *S. pharaonis* hide their eggs in coral crevices, possibly to help guard them against predatory fish (Corner and Moore, 1981; Gutsall, 1989). The flamboyant cuttlefish (*Metasepia* spp.) have been observed to lay their eggs in live rock and coconut shells (in captivity: Grasse, 2014; in the wild: C.L. Huffard, personal observations). As with myopsids and sepiolids, female sepiids possess an ANG which is thought to aid in inoculating their eggs against harmful pathogens (Richard et al., 1979). Similar to sepiolids, sepiids are direct-developing and spend their entire life histories on or near the seafloor (Boletzky, 1987), suggesting that dispersal might be more limited in these orders than in most other cephalopod taxa.

### 2.8. Reproductive Biology in Spirulida

**Figure 7:**
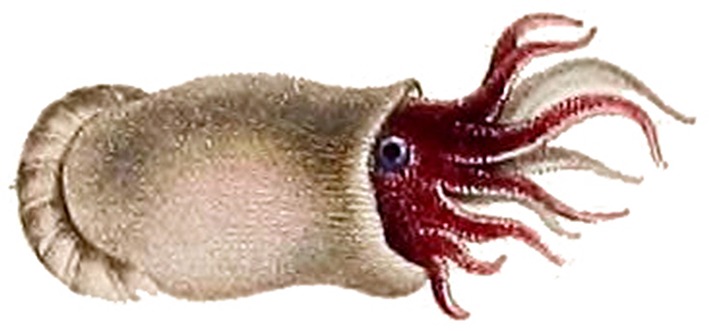
An artist's rendition of the ram's horn squid, *Spirula spirula* (Decapodiformes: Spirulida). This image was drawn by Lesueur in 1807, and was downloaded from the public domain via Wikimedia.

Spirulida (Decapodiformes: Spirulida) is a monotypic order comprising the Ram's horn squid (*Spirula spirula*; Figure 7). Very little is know about the life history or behaviour of this mesopelagic squid, but size profiling of dead specimens caught at different depths and different times of year has led to some insights (Bruun, 1943; Clarke, 1970). This small (up to 45 mm mantle length) and elusive cephalopod is predicted to have an 18–20 month lifespan and is thought to reach sexual maturity at 12–15 months of growth (Jereb and Roper, 2005). Due to observations that the smallest individuals have been caught at the greatest depths (1,000–1,750 m), it has been suggested that females might deposit eggs at the bottom of continental slopes (Jereb and Roper, 2005).

### 2.9. Reproductive Biology in Octopoda

**Figure 8:**
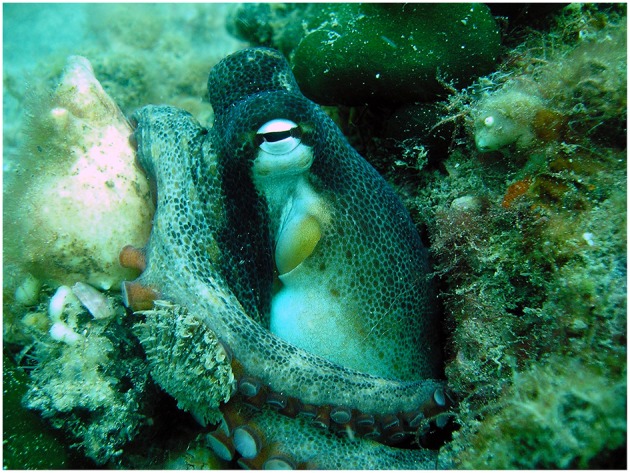
The gloomy octopus, *Octopus tetricus* (Octopodiformes: Octopoda) from Fremantle, Western Australia. Photograph by P. Morse.

**Figure 9:**
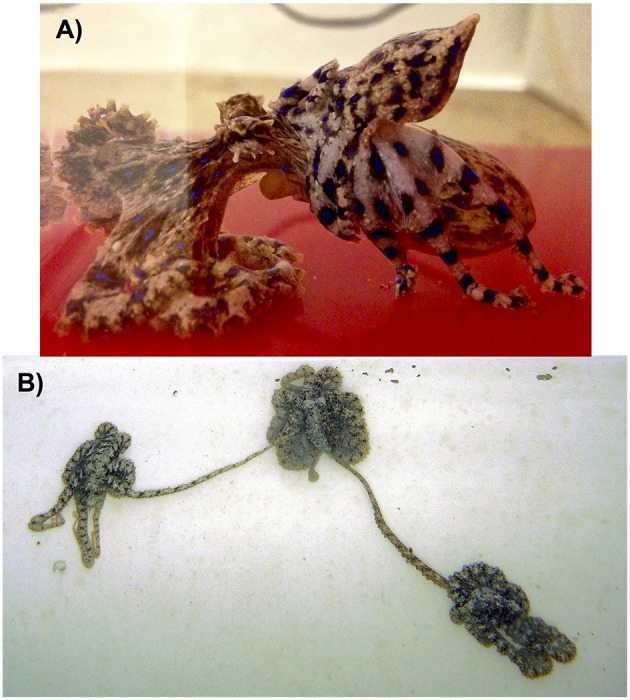
Examples are shown of both the mount **(A)** and reach **(B)** copulation postures displayed by incirrate octopuses. **(A)** A male southern blue-ringed octopus (*Hapalochlaena maculosa*) mounts the female's mantle as he uses his hectocotylised third right arm to transfer spermatophores to the female's distal oviducts. Photograph taken under laboratory conditions by P. Morse; **(B)** Two male algae octopus (*Abdopus aculeatus*) simultaneously mate with a female (centre) by reaching with their hectocotylised third right arms to transfer spermatophores to her distal oviducts. Photograph taken by C. Huffard at Lizard Island, Australia.

The octopods (Octopodiformes: Octopoda) can be broadly divided into two suborders: the incirrate octopods (Octopoda: Incirrata; Figure 8), and the cirrate octopods (Octopoda: Cirrata). Egg fertilisation in the incirrate octopods is always internal (Froesch and Marthy, 1975). The male hectocotylus, which is usually the third right arm (Robson, 1929), terminates in a specialised organ called a ligula (Wells and Wells, 1972). The ligula is composed of erectile tissue in some species (Thompson and Voight, 2003), and it is thought that this structure aids in spermatophore placement and/or removal of competing spermatophores (Voight, 1991; Cigliano, 1995). Males are hypothesised to use the ligula to reach inside the female's mantle aperture and presumably locate one of the two oviducts (Wells and Wells, 1972; Supplementary Video 1). Spermatophores are then passed from the male's terminal organ, which is inside the mantle, through the funnel and into the base of the hectocotylised arm (Wells and Wells, 1972). The spermatophores are carried through a ventral groove in the hectocotylus to the ligula using a wave of contractions along the arm (Wodinsky, 2008). The male then uses the ligula to place each spermatophore at one of the openings to the female's paired oviducts (Wells and Wells, 1972). This process can happen while the male is mounting the female's mantle (e.g., *Eledone* spp. in Orelli, 1962; and *Hapalochlaena* spp. in Tranter and Augustine, 1973; Overath and Boletzky, 1974; Figure 9A), by the male reaching over to the female with the hectocotylus from a distance (e.g., *O. digueti* in Voight, 1991; and algae octopus, *Abdopus aculeatus* in Huffard et al., 2008; Figure 9B), or in a beak to beak mating position with the female at times enveloping the male in her web (LPSO in Caldwell et al., 2015; and occasionally *O. oliveri* in Ylitalo et al., 2019). Males of some species have been observed to use both mounting and reach strategies (*O. cyanea* in van Heukelem, 1966; and *O. tetricus* in Huffard and Godfrey-Smith, 2010), and might use the mounting position more often with females that are unreceptive (P. Morse personal observations with *O. tetricus*).

Osmotic pressure from exposure to seawater (Hanson et al., 1973), and possibly mechanical rupture from the ligula, break open spermatophores and sperm is usually stored as spermatozoa inside the spermathecae of female's oviducal glands (Froesch and Marthy, 1975). However, in several deep-water octopuses (e.g., *Eledone* spp., *G. macrotyla* and *Vulcanoctopus hydothermalis*) spermatangia migrate to the female's ovaries, where fertilisation occurs (Orelli, 1962; Perez et al., 1990; González et al., 2008; Guerra et al., 2012). In the pelagic environment, where the likelihood of encountering a conspecific of the opposite sex may be the relatively low, males in three genera of incirrate octopods, *Argonauta, Tremoctopus* and *Ocythoe*, have hectocotyli that fill with sperm, get broken off and left inside the female's mantle cavity (Laptikhovsky and Salman, 2003). Males of the cirrate octopods do not have a ligula (Villanueva, 1992), and it has not yet been established how copulation occurs.

Fecundity is highly variable among incirrate octopods, and egg count estimates have ranged from approximately 30 in *Bathypolypus arcticus* (O'Dor and Malacaster, 1983) up to 700,000 in *O. cyanea* and *O. tetricus* (van Heukelem, 1966; Joll, 1976). Fecundity within the cirrate octopods has so far only been assessed for *O. grimaldii*, and the maximum fecundity estimated in this species was 3,202 based on follicular sheath and remaining egg counts (Boyle and Daly, 2000). *Opisthoteuthis* spp. lay single eggs continuously throughout their adult life cycle, and there is no indication of parental care within these octopods (Villanueva, 1992; Daly et al., 1998; Boyle and Daly, 2000). Where studied, all incirrate octopods display some form of extended egg care (Joubin, 1933; Tranter and Augustine, 1973; Hanlon et al., 1985; Voight and Grehan, 2000; Huffard and Hochberg, 2005; Miske and Kirchhauser, 2006). Most female incirrate octopods attach eggs to hard substrates, usually inside dens or shelters, where they guard and clean the eggs until hatching (Overath and Boletzky, 1974; Joll, 1976). This maternal behaviour has also been reported in two species of deep-sea octopuses, *Graneledone* sp. and *Benthoctopus* sp. during ROV observations (Voight and Grehan, 2000). These authors suggest, that in an environment with limited substrate, these octopuses aggregate around deep-sea rock outcrops as they begin their brooding phase.

Several other octopod species have ways of carrying their developing eggs with them. *Amphioctopus* spp., *Macrotritopus defilippi, H. maculosa* and *Wonderpus photogenicus*, which all live in sand or silt habitats, carry their eggs in the ventral aboral web, in line of water expelled from the funnel (Tranter and Augustine, 1973; Hanlon et al., 1985; Huffard and Hochberg, 2005; Miske and Kirchhauser, 2006). The pelagic *Boliataena microtyla* carries its eggs and reportedly also their larvae within their arms (Young, 1972). *Tremoctopus* spp. carry their eggs using a calcified material that they secrete from their web and attach to their dorsal arms (Naef, 1928). *Vitreledonella richardi* carries its developing eggs and possibly newly hatched larvae within the female's mantle cavity (Joubin, 1933). The argonauts (Incirrata: Argonautidae) carry their eggs within their shell (Laptikhovsky and Salman, 2003). *Ocythoe* spp. have long winding oviducts, where embryos develop as they pass through (Naef, 1928), making the species of this genus the only known ovoviviparous cephalopods. Upon hatching, octopod larvae are either benthic and resemble their adult forms (e.g., members of the subfamily Bathypolypodinae, Boletzky, 1987; and *H. maculosa*, Tranter and Augustine, 1973) or are planktonic (e.g., many *Octopus* spp., Boletzky, 1987).

### 2.10. Reproductive Biology in Vampyromorphida

**Figure 10:**
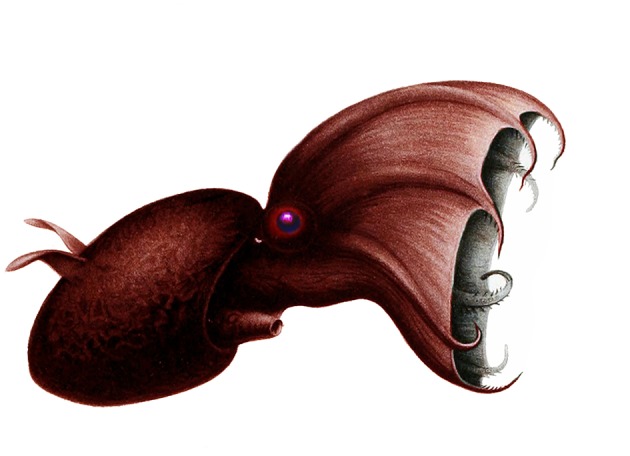
An artist's rendition of the vampire squid, *Vampyroteuthis infernalis* (Octopodiformes: Vampyromorphida). This image was originally designed by Carl Chun in 1910, and was downloaded from the public domain via Wikimedia.

The order Vampyromorphida fits phylogenetically within the superorder Octopodiformes (Allcock et al., 2015), and is represented by only a single extant species, *V. infernalis* (Young et al., 1998; Figure 10). This midwater species occupies the mesopelagic to bathypelagic zones (500–3,000 m, Seibel et al., 1997), and ROV footage has never captured them mating. Therefore, knowledge of reproduction in *V. infernalis* is limited to observations made from dead specimens. *V. infernalis* males lack a hectocotylised appendage, and it is thought that they use their funnel to transfer spermatophores into females' spermathecae, which in this species are two sperm storage pits located beneath females' eyes (Pickford, 1949b). Single *V. infernalis* eggs have been found drifting freely in open waters, suggesting that females might deposit eggs singly into the water column (Pickford, 1949a). Examination of oocyte development and numbers in dead specimens indicate that female *V. infernalis* have multiple spawning events throughout their lifetime, and can have potential fecundity up to 20,711 (Hoving et al., 2015). The paralarvae of *V. infernalis* resemble adults except for that they have a set of oblique fins, which later get reabsorbed as the adult fins grow in (Young and Vecchione, 1999). The paralarvae can swim freely in deep water habitats, however it is not known whether hatchlings have a free-drifting phase before metamorphosing into the described paralarval form (Young and Vecchione, 1999).

## 3. Pre-copulatory Behaviour in Coastal Cephalopods

### 3.1. Female Choice and Male/Male Competition

#### 3.1.1. Loliginidae

Loliginid squid are among the most social of the cephalopods, in that they hunt in shoals and all species mate in large spawning aggregations (Hanlon, 1998). These spawning aggregations are consistently observed to have male-biased OSRs (1.4:1 in Hanlon et al., 2002; 1:1–3:1 in Jantzen and Havenhand, 2003), where there is a high turnover of mates for both males and females and intense male/male aggression over females takes place (Hanlon et al., 1997, 2002; Jantzen and Havenhand, 2003). Currently, females of only one loliginid species are known to be selective of male partners. Wada et al. (2005a) observed *Sepioteuthis lessoniana* in the laboratory and reported that females rejected more than half of copulations attempted by small subordinate males, but rather chose to copulate in 95% of attempts by larger, more dominant males. Hanlon et al. (1994) provided an excellent account of different body patterning and postures employed by male *Loligo vulgaris* within spawning assemblages. These authors suggested that males of this species use courtship in the forms of both chromatophore patterning and by displaying enlarged testes, which are visible in this species through the mantle, to females. However, additional field and laboratory observations of mating behaviour in *Loligo* spp. have suggested that females are likely to accept copulations with all attempting males (Hanlon et al., 2002; Shaw and Sauer, 2004), which questions the need for courtship behaviour. It is possible that, rather than for courtship, these body patterns and testis displays are used for sex identification within loliginid mating systems.

In both *Loligo* and *Sepioteuthis*, male/male aggression and dominance hierarchy greatly influence copulatory success among males (Hanlon et al., 1994, 1997, 2002; Jantzen and Havenhand, 2003; Wada et al., 2005a). Females of these genera usually arrive at spawning grounds already with a paired consort male, and lone large males that are already waiting at egg-laying sites, frequently challenge paired males for consort status (Hanlon et al., 1997, 2002; Jantzen and Havenhand, 2003). These challenges take the form of intense visual signalling and occasionally fin beating. Both Hanlon et al. (2002) and Jantzen and Havenhand (2003) report high turnovers of consorts in *L. vulgaris* and *S. australis*, respectively. Additionally, smaller “sneaker” males attempt sneaker copulations with already paired females, by quickly moving in between females and consort males and attempting to mate with females in a head to head position (Sauer et al., 1997; Hanlon et al., 2002). Sneaker males time their attempts for when females are about to deposit an egg capsule, and place spermatophores either onto females' arms or directly on egg capsules (Hanlon et al., 2002). Jantzen and Havenhand (2003) observed some *S. australis* sneaker males to mimic female body patterns in order to obtain sneaker copulations without prompting aggression from consort males.

Consort males in both genera place spermatophores internally, close to the opening of females oviducts, however this happens in a “male parallel” position in *Loligo* spp. (Hanlon, 1998), while *S. australis* are observed to most often do this in an upturned position (Jantzen and Havenhand, 2003). Hanlon et al. (2002) report that *L. vulgaris* females arrive at spawning sites already having sperm in their receptacles from what are thought to be from previous head to head copulations. It is likely that females of both genera will copulate with males opportunistically outside of spawning aggregations, and store sperm until future egg depositions. Although females of most loliginid species have not been observed to be selective about which males they copulate with (Hanlon et al., 1997, 2002; van Camp et al., 2004; cf. Wada et al., 2005a), the high frequency of multiple copulations between egg laying intervals (Hanlon et al., 2002; Jantzen and Havenhand, 2003), differential male mating strategies with different methods of sperm placement (Hanlon et al., 1997, 2002), females' capacity to store sperm and to possibly be selective about which sperm they use during external fertilisation (Hanlon et al., 2002; Shaw and Sauer, 2004) all suggest that sperm competition, and conceivably post-copulatory female choice could greatly influence male reproductive success in loliginid mating systems (addressed in section 4.2).

#### 3.1.2. Sepiidae

Cuttlefish have highly promiscuous mating systems, and as mentioned in the previous section females spawn multiple times over one or two breeding seasons (Le Goff and Daguzan, 1991; Gabr et al., 1998). Both *Sepia apama* and *S. latimanus* are known to have spawning aggregations in which males congregate around egg-laying sites in order to attempt copulations with spawning females (up to six individuals in *S. latimanus*, Corner and Moore, 1981; up to 1000s of individuals in *S. apama*, Hall and Hanlon, 2002). Field observations at these spawning sites have revealed detailed accounts of natural reproductive behaviour in these species.

As with loliginid squid, the OSR observed at wild cuttlefish spawning assemblages are always male biased (4:1–11:1 in *S. apama*, Hall and Hanlon, 2002; ~3:1–4:1 in *S. latimanus*, Corner and Moore, 1981), consistent with both field and aquarium observations of females copulating with multiple males between egg-laying intervals (aquarium observation of *S. officinalis*, Adamo et al., 2000; and *Sepiella japonica*, Wada et al., 2006; field observations of *S. latimanus*, Corner and Moore, 1981; and *S. apama*, Hall and Hanlon, 2002) and intense male/male aggression over females (Corner and Moore, 1981; Adamo et al., 2000; Schnell et al., 2015). However in contrast to loliginid squid, female cuttlefish are reported to frequently reject male copulation attempts (Corner and Moore, 1981; Adamo et al., 2000; Hall and Hanlon, 2002; Schnell et al., 2015). Notably, wild *S. apama* females have been observed to reject 70% of male copulation attempts and only 3% of all copulations were forced (Hall and Hanlon, 2002), emphasising that pre-copulatory female discretion of males plays an important role in this mating system.

Within *S. apama* spawning sites, larger males typically compete with each other, using moderate physical contact and occasional biting, to gain consort status with females, to which they then transfer multiple spermatophores and mate guard (Hall and Hanlon, 2002). Meanwhile, smaller, lone males either try to locate unguarded females or attempt sneaker copulations with already-paired females (Norman et al., 1999; Hall and Hanlon, 2002). Sneaker copulations in cuttlefish mating systems can be defined in three ways: (i) the sneaker male either overtly follows a guarded female and copulates with her while the consort male is distracted; (ii) the sneaker male can copulate with the guarded female while concealed from the consort male's view, such as by hiding under rocks where females lay eggs; or (iii) similar to *S. australis*, sneaker males can mimic female chromatophore patterning while approaching and mating with the female, to avoid aggression from the consort males (Norman et al., 1999; Hall and Hanlon, 2002). Male *S. plangon*, which do not spawn in aggregations, have also been observed to display female patterning on one half of the body that is exposed to a nearby male, while showing typical male patterning to a female with their other half of the body (Brown et al., 2012).

Field observations of *S. apama* have indicated that females can accept or reject copulations with males regardless of size or mating strategy. Hall and Hanlon (2002) reported that females often rejected large males to later accept copulations with relatively smaller males. Consort males gain more copulations with the females they guard (Hall and Hanlon, 2002; Naud et al., 2004), and this is intuitively an advantageous strategy as males compete intensely for this role. However, small males might still achieve a competitive copulatory success overall, by investing less time per female and thereby being able to copulate with more females. A variety of chromatophore and postural displays are very common during pre-copulatory behaviour in cuttlefish (Corner and Moore, 1981; Boal, 1997; Norman et al., 1999; Adamo et al., 2000; Hall and Hanlon, 2002; Brown et al., 2012; Schnell et al., 2015; Kubodera et al., 2018). In some cases these displays have been suggested as means of courtship (Corner and Moore, 1981; Kubodera et al., 2018). However, there is no direct evidence of females preferentially mating with particular males that display different intensities of these displays, leading some authors to suggest that visual displays, observed during cuttlefish mating interactions, might be used for signalling agonistic intent and in sex recognition (Boal, 1997; Hall and Hanlon, 2002; Schnell et al., 2015).

During laboratory choice trials, *S. officinalis* females also showed no preference for male size or social hierarchy. However, females interestingly spent more time with males that had copulated more recently (Boal, 1997). This finding probably suggests one of two things: either (a) That females show preference for a male trait or behaviour based on chemical or visual cues that have not yet been measured (see section 3.3); or (b) That females can discern males' mating history, possibly based on chemical cues, and are attracted to males that have already established a high copulatory success. If the latter case applies, then there could be a selective advantage for females to prefer males with higher copulatory success, because this is likely to result in them having sons which also have higher copulatory success than competing males. In this way a female preference for male promiscuity could be reinforced through achieving more grandchildren, and this would lead to a *Fisherian* run-away process (Fisher, 1930).

Overall, the fact that there is intense male competition for females during spawning and that female cuttlefish frequently reject male copulation attempts, presumably based on cues other than size or hierarchy, support that female choice plays an important role in the differential reproductive success of male cuttlefish. However, it is still not certain what criteria females might use to discern between potential mates. Also, the details of female sperm storage, external fertilisation and vigilant mate guarding by consort males leading up to egg deposition all suggest that the timing and order of sperm placement are likely to influence the resulting fertilisation patterns (see section 4.5).

#### 3.1.3. Octopodidae

Octopuses are different from cuttlefish and squid in that they are mostly solitary animals, with little to no social interactions outside of agonistic disputes over den space or mates (Cigliano, 1993; Huffard et al., 2008), cannibalism (Hanlon and Forsythe, 2008) and predominantly opportunistic copulations (Young, 1962; Anderson et al., 2003; cf. Huffard et al., 2008). Within mating systems that have been observed in the field, OSR is generally more balanced than is common in decapods (1:1–3.5:1 in *Abdopus aculeatus*, Huffard, 2005; 0.34:1–1.8:1 in *Octopus hubbsorum*, Lopez-Uriarte and Rios-Jara, 2009). This might suggest that pre-copulatory choice could be important for both male and female mate selection within this family, and is consistent with observations that females of at least three species can initiate copulations with males (*O. cyanea*, Wells and Wells, 1972; *Hapalochlaena lunulata*, Cheng and Caldwell, 2000; and *H. maculosa*, Morse et al., 2015). As mentioned previously, all members of the Octopodidae family are terminal spawners with the exceptions of *O. chierchiae* (Rodaniche, 1984) and LPSO (Caldwell et al., 2015).

There is limited evidence for sex recognition and courtship in octopuses. Cheng and Caldwell (2000) observed *H. lunulata* males to attempt copulations with other males as often as with females, suggesting no form of sex recognition prior to copulation in this species. However, *O. bimaculoides* are able to discriminate between different sexes of conspecifics based on odour cues (Walderon et al., 2011). It has been suggested that some octopuses use behavioural cues for sex recognition and possibly courtship. Packard (1961) suggested that male *O. vulgaris* might display their proximal suckers, which are sexually dimorphic and bigger on males in this species, to signal their sex and obtain copulations with females. However, in a follow-up study, males of this species were not observed to display their enlarged suckers to females during laboratory copulations, and therefore there would have been no opportunity for females to assess this trait (Wells and Wells, 1972). Voight (1991) has suggested that the ligula might be used in courtship and influential to male copulatory success. Ligulae have species-specific morphology among octopuses, and Voight (1991) reported male *O. digueti* to display their ligulae to females and make contact with females using their ligulae prior to copulation. However, this study stated that no evidence of true courtship was found. A tactile phase leading up to copulation has also been noted within in pairs of *O. vulgaris, O. cyanea* and *O. tetricus* during laboratory observations (Wells and Wells, 1972; Morse, 2008), and it is possible that this behaviour enables female assessment of males' ligulae. Both *A. aculeatus* and *Amphioctopus marginatus* males have been observed in the field to display different chromatophore patterns to females before copulation (Huffard, 2007; Huffard and Godfrey-Smith, 2010), and in the case of *A. aculeatus*, males approached conspecifics differently depending on the colour pattern displayed by the approached individual. In addition to chromatophore displays, *A. aculeatus* pairs have been observed to synchronously perform a mantle bounce behaviour and females of this species have been observed to change postures to what is called a “DACT display” prior to copulation (Huffard, 2007).

It is possible that some of these behaviours are means of sex and/or species recognition but it is not clear if they are methods of courtship. Females of at least five species of octopus are known to frequently resist and/or reject male copulation attempts in laboratory conditions (*O. cyanea*, Wells and Wells, 1972; *O. digueti*, Voight, 1991; *O. tetricus*, Morse, 2008; *O. bimaculoides* Mohanty et al., 2014; and *H. maculosa* Morse et al., 2015). However, no investigations have yet successfully compared female receptivity to varying forms or intensities of the above traits and behaviours. Additionally, most observations of octopus reproductive behaviour have taken place in the laboratory, where artificial measures of OSR, and confined spaces that limit females' ability to reject copulations, make it difficult to accurately assess potential female preferences and/or which males will achieve higher copulatory success within natural mating systems.

Octopuses known to aggregate in the wild share certain behavioural characteristics in common. *A. aculeatus* (Huffard et al., 2008), LPSO (Caldwell et al., 2015) and *O. tetricus* (Scheel et al., 2016) show specific chomatophore and postural displays to conspecifics, and males and females occupy adjacent dens which facilitates repeated copulations among pairs. Of these three species, male *A. aculeatus* also exhibit high levels of aggression to compete for mate guarding status, denning proximity, and repeated copulations with larger females (Huffard, 2007; Huffard et al., 2008, 2010). Like in loliginid squids, both male and female *A. aculeatus* engaged in opportunistic copulations while foraging away from their dens, and smaller males attempted to gain sneaker copulations with guarded females by camouflaging themselves or hiding behind rocks to not instigate aggression from the guarding males (Huffard et al., 2008). In this species, females were observed to accept copulations with nearly all males. However, due to competition among males, large mate guarding males obtained higher copulation rates within the studied populations (Huffard et al., 2008). Like in loliginid squid and cuttlefish, the high levels of female promiscuity, sperm storage and mate guarding all suggest, that in addition to differential copulatory rates, sperm competition most likely plays an influential role in male reproductive success within shallow-water octopus mating systems (see section 4.6).

### 3.2. Differential Copulatory Success in Females

Currently, male preference of females and differential female copulatory rates have not been extensively noted within loliginid squid or cuttlefish taxa. One study reported that younger *S. australis* females laid more eggs than older females during 1 month of observations in aquaria (van Camp et al., 2005). These authors have suggested that this might signify male preference in this species toward younger females. However, females' capacity for sperm storage and intermittent egg laying among loliginid squid (Shaw and Sauer, 2004; Buresch et al., 2009) means that many of the females might not have finished laying eggs during the duration of this study, and so this limits direct evidence to support the theory of male choice in this species. Among the cuttlefish, male *S. apama* have been observed to preferentially attempt copulations with unfamiliar females (Schnell et al., 2015). However, this observation may have been indicative of the males withholding spermatophores from females with which they had already mated, and might not necessarily result in differential copulatory rates among females within this mating system.

There are however some indications of male choice for females in octopuses. Field observations of *A. aculeatus* have observed males to preferentially mate guard and copulate more with larger females, which are likely to have a higher egg-laying capacity than smaller females (Huffard et al., 2008). Males of this species were also observed to have longer bouts of male/male aggression over larger females, however were more likely to engage in competitive bouts over medium sized females which are less likely to soon be usurped by other larger males (Huffard et al., 2010). Similar observations have been made of *O. bimaculoides* in the laboratory, where higher levels of male-male aggression were reported in the presence of immature females (Mohanty et al., 2014). These authors have hypothesised that a first-male sperm precedence in fertilisation patterns could lead to a greater male investment toward mating with smaller or younger females in some *Octopus* mating systems. However, this hypothesis has yet to be verified through analyses of brood paternities.

Observations of male preference and differential female copulatory success in female octopuses, but not necessarily in the decapods are likely related to differences in OSR among these mating systems (Table 2). In loliginid squid and cuttlefish where OSR is more often to be heavily male biased (Hall and Hanlon, 2002; Jantzen and Havenhand, 2003), it is more likely that males might attempt copulations with every possible female they have access to. In shallow-water octopuses where the OSR is more balanced (Huffard, 2005; Lopez-Uriarte and Rios-Jara, 2009), male selection of females might be an important factor to female reproductive success.

**Table 2:** The precopulatory behaviours of the Loliginidae, Sepiidae and Octopodidae families are summarised below.

**Family**	**Spawning assemblages**	**OSR**	**Pair forming**	**Conditional mating strategies**	**Male agonistic behaviour**	**Mate guarding**	**Courtship**	**Female rejections**	**Female preference**	**Male preference**
Loliginidae	Yes^[1, 2]^	1:1–3:1^[2]^	Temporary (consort males)^[1, 2]^	Yes^[1, 2]^	Yes^[1, 2]^	Yes^[1, 2]^	Suggested in *L. vulgaris*^[12]^	In *S. lessoniana*^[17]^; not reported in other species	For large dominant males in *S. lessoniana*^[17]^	Not reported
Sepiidae	In some species^[3, 4]^	3:1^[3]^–11:1^[4]^	Temporary (consort males)^[4]^	Yes^[4]^	Yes^[4]^	Yes^[4]^	Suggested in two species^[3, 13]^	Yes (frequent)^[4]^	Unclear. Toward males that have mated more recently in *S. officinalis*^[21]^	Possibly toward novel females in *S. apama*^[22]^
Octopodidae	Not reported	0.34:1^[5]^–3.5:1^[6]^	Male and female pairs observed to occupy adjacent dens in four species^[7, 8, 9, 10]^	Observed in *A. aculeatus*^[8]^	In at least two species^[8, 11]^	Observed in *A. aculeatus*^[8]^	Suggested, but not confirmed in many species. Possible courtship through ligula contact^[14]^; and/or chromatophore displays^[15, 16]^	In at least five species^[18, 14, 19, 11, 20]^	Unclear	Toward larger females in *A. aculeatus*^[8]^; and toward younger females in *O. bimaculoides*^[11]^

1 (Hanlon et al., 2002);

2 (Jantzen and Havenhand, 2003);

3 (Corner and Moore, 1981);

4 (Hall and Hanlon, 2002);

5 (Lopez-Uriarte and Rios-Jara, 2009);

6 (Huffard, 2005);

7 (Yarnall, 1969);

8 (Huffard et al., 2008);

9 (Caldwell et al., 2015);

10 (Scheel et al., 2016);

11 (Mohanty et al., 2014);

12 (Hanlon et al., 1994);

13 (Kubodera et al., 2018);

14 (Voight, 1991);

15 (Huffard, 2007);

16 (Huffard and Godfrey-Smith, 2010);

17 (Wada et al., 2005a);

18 (Wells and Wells, 1972);

19 (Morse, 2008);

20 (Morse et al., 2015);

21 (Boal, 1997);

22 (Schnell et al., 2015). Taxonomic families and the sequence they are presented in are based on phylogenies described in Allcock et al. (2015).

### 3.3. The Roles of Signalling and Sensory Ecology in Precopulatory Mate Choice

#### 3.3.1. Visual Signalling

Cephalopods possess a unique system of neurally controlled chromatophores, leucophores, iridiophores and dermal muscles that allow them to rapidly change the colour, tone, pattern and texture of their skin (Packard and Hochberg, 1977; Mäthger and Hanlon, 2007). This ability enables cephalopods to employ impressive crypsis behaviours for defence against potential predators (Huffard, 2006; Krajewski et al., 2009; Staudinger et al., 2011). Additionally, several studies have identified cephalopods to use these pattern-changing abilities as a means of intra-specific signalling (Hanlon et al., 1994; Boal et al., 2004; Palmer et al., 2006). As mentioned above, visual displays using various chromatophore patterns have been observed in spawning assemblages of loliginid squid and cuttlefish, as well as in pre-copulatory behaviours of octopuses (Corner and Moore, 1981; Hanlon et al., 1994; Hall and Hanlon, 2002; Huffard, 2007; Huffard and Godfrey-Smith, 2010; Schnell et al., 2015). It is so far postulated that visual signals might aid in sex and species recognition, and for displaying agonistic intent between con-specifics (Boal, 2006; Scheel et al., 2016). However, no studies so far have shown a conclusive response of opposite sex receivers to these signals, which leaves the role of visual signalling in courtship unclear.

An important aspect of visual signalling in cephalopods is that most studied taxa are not able to discriminate between different wavelengths of light like in human colour vision (Messenger et al., 1973; Mäthger et al., 2009), but rather are sensitive to the angles in which light is travelling (Moody and Parriss, 1961; Saidel et al., 1983; Shashar et al., 1996). This is termed polarisation-sensitivity, and is common amongst invertebrates and has also been reported in some birds and fish (Cronin et al., 2003). Polarisation-sensitivity is considered especially useful in deep-water environments where the wavelength spectrum of light decreases with depth but properties of polarised light remain intact (Shashar and Cronin, 1996). The ability to discriminate polarised light properties likely helps cephalopods with both navigation and in locating crustacean prey-items that have highly polarised exoskeletons (Shashar and Cronin, 1996). However, cephalopods are also able to change the polarised patterns reflected from their skin using their chromatophores and iridiophores (Shashar et al., 1996; Boal et al., 2004). Because cephalopods use skin patterning for visual signalling (Palmer et al., 2006), are polarisation-sensitive (Moody and Parriss, 1961) and have the ability to alter the polarised patterns reflected from their skin (Shashar et al., 1996), this presents the likely possibility that that cephalopods might have the capacity to use polarised signalling as a means of intra-specific communication, imperceptible to the human eye (Mäthger et al., 2009).

Evidence for use of polarised signalling as a communication channel in cephalopods is still very limited. In the laboratory, *S. officinalis* responded differently to their own mirror image depending on whether or not the mirror distorted the reflectance of polarised light (Shashar et al., 1996), and female *S. officinalis* have been observed to display more polarised patterns than males (Boal et al., 2004). However, neither the quantity nor the nature of these displays differed in response to the number or sex of conspecifics viewed by the displaying female (Boal et al., 2004), making it unclear what type of information might be sent or received through polarised signals and what benefit these signals might have for the signaler or receiver. At present, no studies have yet incorporated imaging polarimetry within a context of investigating mate choice or potential courtship. As visual signalling has been observed as an important component of pre-copulatory behaviour in studied cephalopods, the further integration of imaging polarimetry within field or laboratory mate choice studies might be an interesting topic to explore and could reveal substantially more information about cephalopod communication.

#### 3.3.2. Chemoreception

Cuttlefish, squid, and octopus can sense chemical stimuli both from a distance using olfactory organs close to the eyes, and upon contact with objects using chemoreceptor cells located on the lips and suckers (Budelmann, 1996). Distance chemoreception between conspecifics has not yet been investigated within the Loliginidae, however at least some species have the capacity to obtain information from chemical stimuli in the water (Lucero et al., 1992). Tactile chemoreception of conspecific eggs has been investigated within *D. pealei*, and it is suggested that a pheromone present in egg capsules of this species triggers males to engage in male/male agonistic behaviour to compete over females (Buresch et al., 2003; King et al., 2003). This mechanism may be partially responsible for the synchronised spawning assemblages within loliginid taxa (Buresch et al., 2003; King et al., 2003). Distance chemoreception has been investigated in slightly more detail among the cuttlefish. *Sepia officinalis* increase ventilation rates when exposed to seawater containing odour from conspecifics, suggesting that this species can detect other members of its species based on chemical stimuli from a distance (Boal and Marsh, 1998). However, *S. officinalis* does not display any change in approach behaviour based solely on odours from conspecifics of different sex or mating history (Boal and Golden, 1999). Therefore, it is currently not supported that distance chemoreception would play a role in sex identification or mate choice in this species. However, it has not yet been assessed whether chemical cues might influence female receptivity to approaching males.

Distance chemoreception could play a role in the mating system of at least two octopus species. Laboratory trials with *O. bimaculoides* revealed that this species can detect conspecifics based on odour cues, and that ventilation rates of individuals were different depending on the sex of conspecifics that were detected (Walderon et al., 2011). Similar studies with *H. maculosa* found that the change in female ventilation rates in response to male odours correlated with agonistic behaviour and the probability that the female would reject a copulation attempt from the detected male (Morse et al., 2017). Therefore, distance chemoreception might enable some octopuses to determine the sex of conspecifics, and possibly to locate and/or discriminate between potential mates. Octopuses also possess many more chemoreceptors per sucker than decapods (10,000 cells per sucker in octopuses compared to ~100 cells in cuttlefish suckers, Budelmann, 1996). This is likely related to the way in which octopuses reach into holes and crevices while foraging for food (Budelmann, 1996). However, a neurological study in *O. vulgaris* has also identified that the olfactory lobes, responsible for processing the sensory of chemical stimuli, are integrated with parts of the brain that regulate signal molecules involved in reproductive behaviours as well as feeding (Polese et al., 2015). As mentioned above, a tactile phase prior to copulation has been observed in *O. vulgaris* and *O. cyanea* (Wells and Wells, 1972), *O. digueti* (Voight, 1991) and *O. tetricus* (Morse, 2008). It is feasible that because octopuses have well-developed tactile chemoreception, that this could be used by some species to identify species, sex or possibly relatedness and/or quality or potential mates. As yet, the role of tactile chemoreception in mate choice has not been investigated within any cephalopod mating system.

## 4. Post-copulatory Sexual Selection in Coastal Cephalopods

### 4.1. The Role of Sperm Competition in Sexual Selection

The aspects of female promiscuity and sperm storage strongly suggest that post-copulatory processes take an influential role in sexual selection within cephalopod mating systems. The previous section addressed how different traits or behaviours can lead to differential copulatory rates within species. However, reproductive success is based on the quantity of alleles passed on to future generations, and in highly promiscuous mating systems where females store sperm from multiple males in between egg laying intervals, copulatory rates alone will not necessarily determine the reproductive success of individuals. The differential fertilisation success between males that have copulated with the same female is referred to as sperm competition (Parker, 1970). Sperm competition can impact the relative reproductive success of males if certain morphological traits or behaviours can help some males to achieve increased fertilisation success (Parker, 1970). Sperm competition can also affect the reproductive success of females if fertilisation can be biased toward males that are more genetically compatible (Zeh and Zeh, 1996, 1997; Tregenza and Wedell, 2000; Mays and Hill, 2004), or if whichever trait or behaviour used by males to achieve higher fertilisation success can be inherited by their sons (Yasui, 1997; Kokko et al., 2003).

A multitude of factors can affect sperm competition in animal mating systems. Several of these include: The numbers of males contributing sperm to a female (Parker, 1990), the relative contributions of sperm provided by each male (Parker, 1990), removal of previous males' sperm by subsequent male partners (Birkhead and Hunter, 1990), preferential locations for sperm placement (Naud et al., 2005), differential sperm motility (Birkhead et al., 1999), cryptic female choice (CFC) of sperm (Eberhard, 1996), and differential longevity of sperm and/or stratification of sperm within sperm storage receptacles that can lead to differences in fertilisation success based on the order of copulation by competing males (Birkhead and Hunter, 1990; Naud and Havenhand, 2006; Squires et al., 2015; Hirohashi et al., 2016). The current understanding of how sperm competition might impact cephalopod mating systems is still in its infancy. However all of the above mechanisms could potentially influence the relative fertilisation success of male cephalopods (Table 3). The following will summarise the current knowledge of sperm competition in cephalopods based on observations of sperm loading, sperm removal, female choice of sperm, sperm morphology and relative paternity patterns.

**Table 3:** Sperm competitive behaviours and evidence for cryptic female choice among five studied families of Cephalopoda are summarised below.

**Family**	**Sperm loading**	**Sperm removal**	**Differential sperm placement**	**Dimorphic sperm**	**Evidence for separation of sperm in female storage organ(s)**	**Evidence for cryptic female choice**	**Known Predictors of paternity**
Loliginidae	Yes^[1, 2]^	Not reported	Yes^[1, 2]^	Yes^[12]^	Distinct switch in embryo paternity along *L. reynaidiii* egg strings^[13]^; mating plugs in *D. plei*^[14]^	Female *D. pealei* can eject unwanted spermatophores, and possibly influence paternity by delaying egg deposition^[2]^	Higher copulatory rates^[1, 2]^; internal spermatophore placement^[1]^; CFC^[2]^; and interval between copulation and egg deposition^[2]^
Idiosepiidae	Yes^[3]^	Not reported	Not reported	Not reported	Not reported	Female *I. paradoxus* observed using their buccal mass to remove recently transferred spermatophores^[3]^	Not yet investigated. However, mating chronology^[3]^ and duration^[19]^ are suggested to influence paternity
Sepiolidae	Not reported	Suggested^[7]^	Not reported	Not reported	Suggested due to invaginations of the spermatheca^[8]^	Hypothesised^[16]^	Last male paternity bias^[16]^
Sepiidae	Yes^[4, 5]^	Yes^[8, 4, 9]^	Yes^[11]^	Not reported	Different sperm compositions observed in paired receptacles of *S. apama*. Females also have access to separate spermatangia externally placed by males^[11]^	Preferential sperm use from externally placed spermatangia in *S. apama*^[2]^	External placement of spermatophores^[11]^; CFC^[11]^; and suggestion of bias to recently mated males^[20]^
Octopodidae	Hypothesised^[6]^	Suggested^[10]^	Not reported	Not reported	Evidence suggests that sperm is mixed in the oviduccal glands^[15]^	Female *O. vulgaris* can control release of peptides that influence chemotaxis of sperm^[17]^; possible removal of unwanted spermatophores in *O. oliveri*^[18]^	Paternity has been anecdotally observed to correlate with copulation duration^[21]^; mating chronology^[15, 18]^; terminating member of copulation^[15]^; male size^[18]^ and male relatedness to the female^[15]^

1 (Iwata et al., 2005);

2 (Buresch et al., 2009);

3 (Sato et al., 2016);

4 (Hall and Hanlon, 2002);

5 (Wada et al., 2006);

6 (Morse et al., 2015);

9 (Squires et al., 2013);

8 (Hanlon et al., 1999);

9 (Wada et al., 2010);

10 (Cigliano, 1995);

11 (Naud et al., 2005);

12 (Iwata et al., 2018);

13 (Naud et al., 2016);

14 (Saad et al., 2018);

15 (Morse et al., 2018a);

16 (Squires et al., 2015);

17 (De Lisa et al., 2013);

18 (Ylitalo et al., 2019);

19 (Sato et al., 2016);

20 (Hanlon et al., 2005);

21 *(Morse, 2008). Taxonomic families and the sequence they are presented in are based on phylogenies described in Allcock et al. (2015)*.

### 4.2. Sperm Competition in Loliginidae

Sperm competition has been investigated relatively more thoroughly in loliginid squid than in other cephalopod taxa. The current literature suggests that males employ sperm loading (Hanlon et al., 1997, 2002; Jantzen and Havenhand, 2003), but that also sperm placement (Iwata et al., 2005), the interval between copulation and egg deposition (Buresch et al., 2009; Hirohashi et al., 2016), spermatophore morphology (Iwata et al., 2018) and possibly CFC of stored sperm (Shaw and Sauer, 2004; Buresch et al., 2009) can all influence fertilisation patterns. Loliginid males compete for consort status with females that they copulate with repetitively and guard from rival males (Hanlon et al., 1997, 2002; Jantzen and Havenhand, 2003). This suggests that sperm loading may be important for male fertilisation success. Sperm loading has been confirmed in laboratory paternity experiments with *Heterololigo bleekeri* (Iwata et al., 2005) and *Doryteuthis pealei* (Buresch et al., 2009) where higher copulatory rates, and presumably more spermatophores transferred, resulted in higher male fertilisation success. These studies also found that paternities were biased to males that copulated with females in a parallel position that enabled internal placement of spermatophores (Iwata et al., 2005; Buresch et al., 2009), however that sperm from sneaker males, placed in the seminal receptacles, had greater longevity (Hirohashi et al., 2016).

A recent investigation of spermatophore morphology in the chokka squid, *Loligo reynaudii* revealed that large and small males, which typically employ consort and sneaker mating strategies, respectively, have distinct types of spermatophores specialised for their mating strategy and method of sperm transfer (Iwata et al., 2018). However, genotyping of *L. reynaudii* egg strings obtained from wild spawning assemblages have confirmed that paternity was typically biased to the male observed guarding the female at the time of collection (Naud et al., 2016). Interestingly, microscopy analyses of female seminal receptacles in *D. plei* have revealed that everted spermatophores can act as a mating plug, limiting females' ability to access stored sperm from competing males the female had previously mated with for up to 48 h (Saad et al., 2018). These authors hypothesised that this could be a sperm competitive strategy, advantageous for sneaker males that can intercept females between copulations with consort males and egg deposition. Overall, paternity bias to higher copulatory rates, the parallel mating strategy and mate guarding males suggest that consort males will generally achieve higher fertilisation success within the mating systems of loliginid squids, and this further explains both why males compete vigorously for this mating strategy (Hanlon et al., 1997, 2002; Jantzen and Havenhand, 2003) and why sneaker males have to metabolically invest so heavily into producing competitive sperm (Hirohashi et al., 2016; Iwata et al., 2018; Saad et al., 2018).

Female loliginid squid may also have the capacity to influence the paternities of their egg capsules post-copulation. A female *D. pealei* has been observed to eject spermatophores from her mantle after a forced copulation (Buresch et al., 2009), and these authors also identified that the interval between copulation and egg deposition greatly affects egg capsule paternity. When females of this species laid egg capsules within 40 min of copulation, the egg capsules were fertilised mostly by older sperm from previous male partners. However, after 140 min, paternity of egg capsules was biased to the most recent male to have copulated with the female. Additionally, Naud et al. (2016) observed a distinct switch in embryo paternity along *L. reynaudii* egg strings, suggesting that females of this species might have been using different males' sperm for egg fertilisation in non-random patterns. If females can reject spermatophores and presumably can choose when to lay egg capsules (Buresch et al., 2009), then these observations combined with females' capacity to be selective of stored sperm use during external fertilisation (Shaw and Sauer, 2004; Naud et al., 2016), suggest that female loliginids might be capable of controlling which males' sperm fertilise their egg capsules.

### 4.3. Sperm Competition in Idiosepiidae

Owing to their ease of husbandry in captivity (Nishiguchi et al., 2014) there is a currently growing literature investigating the mating system of idiosepiids. Relatively recent laboratory investigations have revealed novel insights to processes of post-copulatory mate choice within the Japanese pygmy squid (*Idiosepius paradoxus*), suggesting that both sperm competition and CFC might be prevalent within the mating system of this taxon (Sato et al., 2013, 2016). These studies indicated that both larger males and males who copulated for longer with females, externally transferred more spermatophores to the base of females' arms during mating (Sato et al., 2016). However, females of this species were observed to use their buccal masses to remove spermatophores from larger males, favouring the retention of spermatophores by males who copulated with them for longer durations (Sato et al., 2016). Additionally, these females were more likely to be selective of transferred spermatophores during subsequent copulations, suggesting possible female post-copulatory trade-up behaviour (see Pitcher et al., 2003) in this species (Sato et al., 2013). Such findings emphasise that both mating chronology and mating duration may be of critical importance to paternal success within this idiosepiid, but interestingly analysis of brood paternity in *I. paradoxus* did not reveal any fertilisation bias to recently mated males (Sato et al., 2016).

### 4.4. Sperm Competition in Sepiolidae

Similar to the idiosepiids, the amenability of some sepiolids to the laboratory environment has prompted recent and novel investigations of sperm competition within this family. Squires et al. (2013) observed that male *Euprymna tasmanica* repeatedly contract their mantle “pumping” while copulating with females. Interestingly, this study found that the frequency of this male pumping behaviour increased with number of copulations the female recently had, and these authors hypothesised that this pumping behaviour might indicate male removal of accessory seminal fluids left behind by competing males. This correlation of pumping behaviour and female mating history implies that male *E. tasmanica* were able to assess the amount of sperm stored by females. However, in this study the males also increased the frequency of their pumps even when they had recently mated with the same female, suggesting that they might not be able to recognise their mates or whether the stored sperm was their own (Squires et al., 2013).

Analysis of brood paternities in *E. tasmanica* revealed that multiple paternity is very common (Squires et al., 2014) but that paternity is frequently biased to the last males to mate with the female (Squires et al., 2015). The spermatheca of female *E. tasmanica*, the site of sperm storage, has been described as “invaginated” and “highly pocketed” (Norman and Lu, 1997; Squires et al., 2013), suggesting that females may be able to partition different males' sperm and use it selectively to fertilise their eggs through CFC. Despite indications of a last-male sperm bias and the potential capacity of females to partition male sperm, evidence of female choice either in the forms of pre-copulatory trade-up behaviour or CFC have not yet been observed in this family.

### 4.5. Sperm Competition in Sepiidae

Sperm competition behaviours have been relatively well-documented within cuttlefish taxa during observations in both the laboratory and field. Cuttlefish males perform sperm removal (Hanlon et al., 1999; Wada et al., 2005b, 2006, 2010), some degree of sperm loading (Hall and Hanlon, 2002; Wada et al., 2006) and non-random patterns of fertilisation have been observed within females' egg masses (Naud et al., 2005). As mentioned in section 3.1.2, cuttlefish males compete for consort status with females whom they guard from rival males and occasionally pass more than one spermatophore (Corner and Moore, 1981; Adamo et al., 2000; Hall and Hanlon, 2002). Copulating multiple times with the same female and mate guarding suggests that relative sperm contributions are likely important for fertilisation success within these mating systems. Additionally, Hanlon et al. (1999) observed three stages of copulation in *Sepia officinalis*. The first stage, which is the longest, is spent using the siphon to flush water over females' buccal areas, likely attempting to remove sperm from either the seminal receptacles or from spermatangia left on females' exterior. The second stage consisted of males transferring new spermatophores to females' buccal membranes, and the third stage was spent breaking open the newly placed spermatophores and ensuring spermatangia attachment.

Male sperm removal has also been indicated in *S. apama* (Hall and Hanlon, 2002; Naud et al., 2004). However, Naud et al. (2005) found that water flushing did not reduce the counts of spermatangia found on females' buccal areas in *S. apama*, suggesting that males of at least this species possibly aim to remove sperm specifically from within seminal receptacles. Male *S. lycidas* use arm III to scrape old sperm masses from females' buccal areas, and spend more time doing this if they are not the last male to have copulated with the female (Wada et al., 2010). This same study identified that larger males of this species will also spend more time than smaller males removing sperm. These authors suggest that smaller males might choose to pass spermatophores sooner if copulation might be likely to get interrupted by a larger male. *Sepiella japonica* has also been observed to briefly remove previous males' spermatangia using arm IV (Wada et al., 2006). However, these authors suggest that male *Sepiella* spp. might invest more time toward sperm loading than removal compared to *Sepia* spp. In this study, *S. japonica* males were observed to display intense mate guarding and in most cases would transfer more than one spermatophore to guarded females.

Currently, cuttlefish fertilisation patterns have been investigated only within wild populations of *S. apama*. Naud et al. (2004) found that males of all sizes and mating strategies had equal fertilisation success among eggs sampled from spawning areas. However, paternity comparisons within individual females' egg clutches were biased to spermatangia left on females' mantles and buccal areas in Naud et al. (2005). This suggests that it might be advantageous for males to copulate with females shortly before egg deposition and to place sperm externally on females rather that in the seminal receptacle. This pattern is supported in a study by Hanlon et al. (2005), in which a female-mimicking sneaker male that achieved a copulation with a female directly prior to egg deposition, was observed to fertilise that egg. If there is a last-male paternity bias to egg fertilisation in *S. apama* this would emphasise the importance of male sperm removal behaviour, and the monopolisation of access to females by consort males near and at egg-laying sites (Hall and Hanlon, 2002).

It is also noteworthy that Naud and Havenhand (2006) discovered that sperm, stored within intact spermatophores, can have longevities up to 2 months. As *Sepia* spp. are intermittent terminal spawners (Rocha et al., 2001), this suggests that females might be able to use stored sperm for future egg fertilisations, and might possibly do so outside of spawning aggregations. Future studies investigating which males' sperm are stored in seminal receptacles versus placed externally as spermatangia might yield further information about sperm competition in this species. Also, the combination of female pre-copulatory choice of males (section 3.1.2) with the suggestion of a last-male paternity bias, presents a question of whether females might assess potential male partners differently based on the types of males they have recently copulated with. Future studies observing female receptivity to sequential males, either in the field or laboratory, might elucidate whether female trade-up behaviour occurs in cuttlefish mating systems.

### 4.6. Sperm Competition in Octopodidae

The mechanisms of sperm competition are much less understood within octopus mating systems. It is probable that males of several species perform sperm loading and sperm removal. However, this has only been formally addressed within one laboratory study (Cigliano, 1995), and currently very few controlled paternity experiments have allowed fertilisation success to be compared among different males (cf. Morse, 2008; Morse et al., 2018a; Ylitalo et al., 2019). Copulation durations are generally much longer in octopuses than in decapods (Joll, 1976; Morse et al., 2018a). Copulations have been observed to last more than an hour in most studied taxa, with the longest copulation being reported as 360 min in laboratory observations of *Octopus tetricus* (Joll, 1976). Field observations of *Abdopus aculeatus* also report males to guard and copulate repeatedly with certain females (Huffard et al., 2008), and laboratory studies with *Hapalochlaena maculosa* have observed males of this species to mate for longer with both unfamiliar females and females that had recently mated with another competing male (Morse et al., 2015). Prolonged copulation durations, mate guarding and multiple copulations with the same females suggest that sperm loading might be an important factor for male fertilisation success. However, it is currently not known whether longer copulation times allow males to pass more spermatophores to females, and/or also might allow males to remove more sperm from previous males, or whether increased copulation time might also be a form of mate guarding whereby the males could be monopolising female opportunities to mate with competing males.

One study, assessing sperm transfer in an unidentified pygmy octopus, found that this species had three phases of copulation, similar to sepiids (Cigliano, 1995). This author suggested that males might use their ligulae to remove competing sperm from females' oviducts during an initial phase of copulation, prior to transferring new spermatophores. Males were also observed to spend more time with the ligula inserted in the female's mantle cavity prior to spermatophore transfer if the female had recently copulated with a different male. However, males spent less time doing this if they were the last males to copulate with the same female. Males could apparently assess females' recent mating history based on the presence or absence of sperm in either the distal portion of females' oviducts or the oviducal glands (Cigliano, 1995). However, it is impressive that males were able to determine if that sperm was their own, as the mechanism enabling them to do this is currently unknown and evidence for mate recognition among octopuses is very limited (Boal, 2006; but cf. possible cases in Caldwell et al., 2015; and Morse et al., 2015).

Four molecular studies within the Octopodidae have so far confirmed multiple paternities within egg clutches of *O. tetricus* (Morse, 2008)*, O. vulgaris* (Quinteiro et al., 2011), *H. maculosa* (Morse et al., 2018a) and *O. oliveri* (Ylitalo et al., 2019). It has so far been postulated that female octopuses might benefit from polyandry due to increased genetic diversity of their offspring (Quinteiro et al., 2011) and the likelihood of reducing fertilisation to related males (Morse et al., 2018a). However, these hypotheses have not yet been empirically tested. As copulation durations are markedly longer in octopuses than decapods (Joll, 1976; Morse et al., 2018a), it remains an interesting question whether female octopuses might be able to influence male fertilisation success by controlling the duration of copulations with different males. Studies of two octopus species have observed females to consistently be the terminating member of copulations, suggesting that they might have control over the length of their copulation time (*O. digueti*, Voight, 1991; and *H. lunulata*, Cheng and Caldwell, 2000). Additionally, a combination of male mating order and whether or not a copulation was ended by the female had a strong effect on paternal success in *H. maculosa* (Morse et al., 2018a). Therefore, female control of copulation time could be a possible form of intra-copulatory mate choice in some species. However, where studied, there is mixed support for the correlation of copulation duration and male fertilisation success (Morse, 2008; Morse et al., 2018a).

Although not yet empirically demonstrated, the reproductive system of female octopuses suggests that CFC may also occur in this family. Female octopuses possess paired, muscular and innervated oviducal glands (Froesch and Marthy, 1975), which they could theoretically use to selectively pump, or block access of sperm to the egg during fertilisation. Additionally, chemoattractant peptides have been found in egg capsules of *O. vulgaris*, that can influence the chemotaxis of male sperm (De Lisa et al., 2013). This suggests that both mechanical and chemical processes might potentially be used by some female octopuses in manipulating the storage or fertilisation success of different males' sperm in their oviducal glands. However, at present there is very little evidence that female octopuses do this (Morse et al., 2018a).

## 5. Conclusions and New Directions for Research

### 5.1. A Summary of Pre- and Post-copulatory Behaviours Warranting Further Investigation Among Well-Studied, Coastal Cephalopods

Currently, the mechanisms of sexual selection are more thoroughly understood within some decapod mating systems than in those of octopuses. The coastal spawning aggregations of *Sepia apama* and loliginid squid have enabled much more detailed investigations within natural settings. Within loliginid mating systems, females of most studied species appear receptive to copulations with every attempting male (cf. *Sepioteuthis lessoniana*, Wada et al., 2005a). However it is strongly suggested that females might be selective of which sperm they use to fertilise egg capsules (Naud et al., 2016). As copulations are usually very quick in these taxa (2–39 s in Hanlon et al., 2002), it might be more parsimonious for females to avoid potential male aggression and the time or energy spent on rejecting males, by being receptive to every copulation and then to control egg capsule paternities post-copulation. Continued observations in the field might be able to further identify the context of both spermatophore rejections and varying intervals between copulation and egg deposition. If females eject spermatophores more or less often with and/or can adjust the timing of egg capsule deposition after copulating with different males that have varying displays, mating strategies or morphologies, then females might use these mechanisms as a form of CFC to bias paternity to genetically fitter and/or more compatible males (Eberhard, 1996; Tregenza and Wedell, 2000).

Within cuttlefish mating systems, females appear highly selective of male partners. It is presently unknown what cues females might use to discriminate between potential males, whether certain males get preferential spermatophore placement in females' seminal receptacles or buccal areas, whether the suggestion of a last-male paternity bias is accurate and whether this consistently leads to increased female selectivity with successive males. It is suggested here that further studies of cuttlefish taxa, either in the field or in large aquaria with male-biased OSR, might provide this information if they can asses the context of different spermatophore placements, compare egg paternities to the order of copulation with genotyped males and compare female-male rejection rates between the first and subsequent males that attempt to copulate with females within egg-laying intervals.

There is still much that can be learned about the processes of mate choice and sperm competition among the octopuses. Further observational studies and/or laboratory choice trials in species where visual courtship displays and female-male rejection are common might unveil whether cues such as ligula morphology or visual displays influence pre-copulatory mate choice in these taxa. Additionally, further paternity comparisons with genotyped candidate fathers (e.g., Morse et al., 2018a) across additional taxa could reveal whether certain types of males gain higher fertilisation success within octopus mating systems, and also whether female brood paternities might be biased toward longer copulation durations, indicating sperm loading, or toward recent males, suggesting the influence of sperm-removal. If sperm loading is identified as an important factor in male fertilisation success, then it will be worthwhile investigating differential copulation durations in species where copulations are frequently terminated by females. This might determine whether females can influence their brood paternities by adjusting copulation times with males that display different morphology or behaviour.

As females of at least two octopus species are suggested to be capable of conspecific sex recognition based on odour cues (Walderon et al., 2011; Morse et al., 2017), it is worthwhile continuing to investigate the role of both distance- and tactile chemoreception within octopus mating systems. Two interesting follow-up questions that could be investigated within laboratory experiments are a) Whether males respond differently to touch or odours from sexually mature vs. immature females; and b) Whether either sex responds differently to touch or odours from novel vs. familiar conspecifics. Answering these questions could help to define the role of chemosensory in octopus social recognition and mate choice behaviours. Additionally, as mentioned in section 3.3.1, visual displays have been reported as part of pre-copulatory behaviour of all loliginids, cuttlefish and octopuses studied in the field (Corner and Moore, 1981; Hanlon et al., 1994; Hall and Hanlon, 2002; Huffard, 2007; Huffard and Godfrey-Smith, 2010; Schnell et al., 2015). However, in order to make sense of these behaviours it is necessary to interpret how these displays are perceived by receiving conspecifics. As most cephalopods are polarisation-sensitive (Moody and Parriss, 1961), yet colour-blind (Mäthger et al., 2009), the further integration of imaging polarimetry into field studies and laboratory mate choice trials is suggested to reveal valuable information about the way cephalopods might communicate within spawning assemblages or in a context of sex identification and/or courtship (Table 4).

**Table 4:** New directions for the research of sexual selection in cephalopods are summarised below. Ten questions warranting further investigation in the near future are presented alongside summaries of potential methodology for addressing them.

**Theme**	**10 questions worth continued investigation**	**Examples of methodology**
Precopulatory Behaviour	How do females of some cuttlefish and octopus species discriminate among male visual displays?	Compare rates of female rejection/receptivity to varying intensities of display. Ideally incorporate imaging polarimetry to quantify and simulate how displays are perceived by the female.
	Do female cuttlefish perform trade-up^[1]^ mate choice behaviour?	Compare rates of female-male rejection among controlled sequential laboratory pairings. Additionally, confirm prevalence of last male paternity using genotyped candidate fathers.
	What are the roles of chemoreception in social recognition and mate choice?	In spp. that cannot visually recognise individuals, assess whether subjects can recognise individuals through distance or tactile chemoreception. Compare ventilatory and/or retreat response to odours and/or touch of familiar vs. novel conspecifics.
	How do males of some octopus species recognise if they were the last male to mate with a female?	Assess whether male octopuses can distinguish the spermatophores/spermatozoa of other males from their own using tactile chemoreception.
	Is sexual selection for sophisticated reproductive behaviours partially responsible for the evolution of complex cognition among cephalopods?	Make a comparative study examining performance on tasks assessing cognitive attributes such as object permanence, working memory, and theory or mind among cephalopod taxa with a variety of reproductive strategies. Use principal component analyses to identify whether particular reproductive dynamics, such as spawning in assemblages, is a predictor of cognitive performance.
Postcopulatory Processes	What criteria influences CFC in cephalopods with external fertilisation?	In controlled laboratory conditions, further identify what factors and/or context (e.g., male phenotype or mating order) lead to higher rates of spermatophore removal and/or delay in egg deposition in spp. where CFC is easily observable (e.g., *D. pealei*).
	How might sperm-attractant peptides influence fertilisation patterns of octopuses?	Use laboratory pairings of genotyped candidate parents, and compare (A) resulting paternity; (B) allelic signatures of sperm remaining in oviducal glands after egg deposition; and (C) concentrations of sperm-attractant peptides in the female reproductive tract at different intervals between copulation with each male and egg deposition.
	Is CFC more common in species where either female-male rejections are rare, or copulations are often forced by males?	Use a meta-analysis to compare presence of CFC behaviour with rates of female rejection and forced copulations among studied cephalopod species.
	Can a “good sperm” hypothesis^[2]^ help to explain widespread polyandry among cephalopods?	Identify whether copulatory rates and/or fertilisation success are correlated among fathers and sons (e.g., heritable) within each laboratory-amenable family.
	Does polyandry help to facilitate inbreeding avoidance?	In spp. with limited dispersal (e.g., *Euprymna* spp. or *H. maculosa*) compare paternal success among genotyped candidate parents having variable but known relatedness to the female.

1 *(Pitcher et al., 2003)*;

2 *(Yasui, 1997)*.

### 5.2. Addressing Widespread Polyandry Among the Cephalopod Class

A common theme amongst all studied cephalopod mating systems is the extremely high level of both male and female promiscuity (Hall and Hanlon, 2002; Hanlon et al., 2002; Huffard et al., 2008; Arnold, 2010; Squires et al., 2013; Morse et al., 2015). Male promiscuity is common within animal mating systems, and can develop easily as an evolutionarily stable strategy (see Smith, 1982) because promiscuity directly increases male reproductive success (Bateson, 1983). Female promiscuity is less commonly reported among species where females do not receive material resources or parental care from the males they mate with, because females have a finite number of eggs they can lay in a lifetime and therefore their reproductive success is typically not limited by the numbers of males they can copulate with (Kodric-Brown and Brown, 1987). Additionally, copulating with lots of different males can be potentially quite costly to females due to the increased risk of injury during copulations (Adamo et al., 2000; Hoving et al., 2010), decreased foraging time (Huffard et al., 2008), increased risk of disease transfer (Thrall et al., 2000), increased energy expenditure (Franklin et al., 2012) and decreased life expectancy (Franklin and Stuart-Fox, 2017). As polyandry appears to be an evolutionarily stable strategy among cephalopods, it is inferred that promiscuous females must achieve some type of selective advantage over non-promiscuous females in order to offset the inherent costs of multiple mating described above.

So far, polyandry in cephalopods has been suggested to benefit females by either helping to overcome potential sperm-limitation (van Camp et al., 2004), increasing the genetic diversity of females' offspring (Quinteiro et al., 2011), and/or optimising offspring quality (Squires et al., 2012; Naud et al., 2016; Morse et al., 2018a). Sperm limitation might be an important factor to female reproductive success in species that have high egg-laying capacities and that might have infrequent encounters with opposite sex conspecifics (e.g., *Architeuthis* spp., Hoving et al., 2004). However sperm limitation probably cannot explain polyandrous behaviour in female cephalopods that have smaller fecundities and that would have the capacity to fertilise all their offspring to one male (e.g., Sepiolidae or *Hapalochlaena* spp.). Offspring diversity probably does increase the fitness of promiscuous females. However, this mechanism alone being the drive for cephalopod polyandry is not consistent with observations of female-male rejections in many taxa, or with observed paternities consistently biased toward particular males (Iwata et al., 2005; Naud et al., 2005, 2016; Morse, 2008; Buresch et al., 2009; Squires et al., 2015; Morse et al., 2018a; Ylitalo et al., 2019) rather than shared more equally between candidate fathers as would be expected in a bet-hedging strategy.

The optimisation of offspring quality appears to be a robust hypothesis for the evolution of polyandry in cephalopod mating systems (Squires et al., 2012; Naud et al., 2016). However, the exact processes for how female promiscuity might lead to enhanced offspring quality still remain unclear. It has been previously hypothesised that nutritional benefits provided from accessory seminal fluids, obtained either through spermatophore consumption or absorption within the female reproductive tract, can help to increase metabolic resources females have available toward producing healthy offspring (Squires et al., 2012; Wegener et al., 2013). A controlled study of *Euprymna tasmanica* has indicated that females of this species, which mated multiply, laid eggs with a higher hatchling to egg mass ratio than females that were only allowed to mate once (Squires et al., 2012). These authors suggested that the added nutritional benefit of receiving extra spermatophores might enable females of some species, particularly ones with internal sperm storage, to maximise their reproductive output relative to maternal investment. However, Squires et al. (2012) also advocated that nutritional benefits likely coincide with indirect, genetic benefits of female promiscuity to provide selective advantages for the widespread polyandry observed among cephalopod taxa.

Postcopulatory fertilisation bias to either reproductively successful males or genetically compatible males are two indirect mechanisms that could also lead to selective advantages for polyandry (Zeh and Zeh, 1996, 1997; Yasui, 1997). However, at present neither scenario has yet been investigated within a cephalopod mating system. Postcopulatory mechanisms might be especially applicable if females either cannot accurately assess male fitness or relatedness during pre-copulatory choice, and/or have limited control of which males they copulate with. In these contexts, polyandrous females could theoretically benefit from accepting sperm from multiple males if differential sperm fertilisation ability, or CFC consistently bias brood paternities to either the fittest or least related males. In the former scenario, if females' offspring are disproportionately sired to males that are innately capable of obtaining a higher fertilisation success, then promiscuous females are also likely to have sons with higher fertilisation success and therefore more grandchildren than non-promiscuous females (Yasui, 1997). This mechanism could potentially be investigated within laboratory paternity comparisons over several generations, and might be supported if copulatory rates and/or fertilisation success are correlated between fathers and their sons. In the case of post-copulatory mechanisms biasing paternity to genetically compatible males, it is possible that female promiscuity is a form of ensuring inbreeding avoidance (see Tregenza and Wedell, 2002). A recent molecular study assessing the relatedness of populations in a holobenthic octopus with limited dispersal revealed high frequencies of close relatives within spawning sites (up to 78% half-half sibling pairs in *H. maculosa*, Morse et al., 2018b). Genomic studies within wild populations across additional cephalopod taxa, and paternity comparisons with known relatedness between mothers and candidate fathers could explain whether inbreeding avoidance might be one of the evolutionary drives for promiscuous behaviour in cephalopods (Table 4).

### 5.3. The Mating Behaviour of Most Cephalopods Is Still Unknown

Finally, it is worth noting again that the bulk of current knowledge for cephalopod sexual selection is still confined to the five families: Loliginidae, Idiosepiidae, Sepiidae, Sepiolidae and Octopodidae. The extreme depths, pelagic environments and specialised nutritional requirements of pelagic and/or deep-sea cephalopod taxa make it difficult to observe them in their natural habitats or maintain them for robust laboratory studies (Hoving et al., 2013). However, at least nautilids appear amenable to aquarium settings (Mikami and Okutani, 1977; Arnold, 2010), and hopefully methods will become available in the future for maintaining other deep-sea or pelagic cephalopod species successfully in the laboratory. Investigating pre-copulatory behaviour and fertilisation patterns of additional cephalopod taxa, either through laboratory rearing or ROV voyages, can likely provide valuable context to the current understanding of sexual selection and behavioural ecology in this unique class of animals.

## Author Contributions

PM compiled the literature and was the primary author. CH provided an expert review of content and revisions to the original manuscript.

### Conflict of Interest Statement

The authors declare that the research was conducted in the absence of any commercial or financial relationships that could be construed as a potential conflict of interest.

## Supplementary Material

Supplementary Video 1 A short copulation in a pair of southern blue-ringed octopus (*Hapalochlaena maculosa*). The male inserts both right arm 2 and his hectocotylus (right arm 3) into the female's right mantle aperture at sec 21, and can be seen removing them at sec 26. This copulation was likely interrupted by the presence of the camera. Video by P. Morse.
